# A Quantitative Systematic Review of Clinical Outcome Measure Use in Peripheral Nerve Injury of the Upper Limb

**DOI:** 10.1093/neuros/nyab060

**Published:** 2021-03-08

**Authors:** Ralph N A Murphy, Heba Elsayed, Sahiba Singh, Jo Dumville, Jason K F Wong, Adam J Reid

**Affiliations:** Blond McIndoe Laboratories, Division of Cell Matrix Biology and Regenerative Medicine, School of Biological Sciences, Faculty of Biology, Medicine and Health, The University of Manchester, Manchester Academic Health Science Centre, Manchester, UK; Department of Plastic Surgery & Burns, Wythenshawe Hospital, Manchester University NHS Foundation Trust, Manchester Academic Health Science Centre, Manchester, UK; Blond McIndoe Laboratories, Division of Cell Matrix Biology and Regenerative Medicine, School of Biological Sciences, Faculty of Biology, Medicine and Health, The University of Manchester, Manchester Academic Health Science Centre, Manchester, UK; Department of Plastic Surgery & Burns, Wythenshawe Hospital, Manchester University NHS Foundation Trust, Manchester Academic Health Science Centre, Manchester, UK; Division of Nursing, Midwifery and Social Work, School of Health Sciences, Faculty of Biology, Medicine and Health, University of Manchester, Manchester Academic Health Science Centre, Manchester, UK; Blond McIndoe Laboratories, Division of Cell Matrix Biology and Regenerative Medicine, School of Biological Sciences, Faculty of Biology, Medicine and Health, The University of Manchester, Manchester Academic Health Science Centre, Manchester, UK; Department of Plastic Surgery & Burns, Wythenshawe Hospital, Manchester University NHS Foundation Trust, Manchester Academic Health Science Centre, Manchester, UK; Blond McIndoe Laboratories, Division of Cell Matrix Biology and Regenerative Medicine, School of Biological Sciences, Faculty of Biology, Medicine and Health, The University of Manchester, Manchester Academic Health Science Centre, Manchester, UK; Department of Plastic Surgery & Burns, Wythenshawe Hospital, Manchester University NHS Foundation Trust, Manchester Academic Health Science Centre, Manchester, UK

**Keywords:** Systematic review, Outcome assessment, Peripheral nerve injury

## Abstract

**BACKGROUND:**

Peripheral nerve injury (PNI) is common, leading to reduced function, pain, and psychological impact. Treatment has not progressed partly due to inability to compare outcomes between centers managing PNI. Numerous outcome measures exist but there is no consensus on which outcome measures to use nor when.

**OBJECTIVE:**

To perform a systematic review in order to describe and classify outcome measures used in PNI.

**METHODS:**

A search of Ovid Medline, Ovid Embase, Allied and Complementary Medicine Database (AMED), and CENTRAL (Cochrane Clinical Trials) was conducted. Randomized control trials (RCTs), cohort studies, and case-controlled and case series (≥5 participants) published from inception of the database until 2019 investigating adult patients with a traumatic upper limb PNI in which an outcome measurement was utilized were included.

**RESULTS:**

A total of 96 studies were included (15 RCTs, 8 case-control studies, 18 cohort studies, 5 observational studies, and the remainder were case series or retrospective reviews). A total of 56 individual outcome measures were identified, utilized across 28 different countries and 7097 patients. Ten core domains were defined: sensory subjective, sensory objective, motor subjective, motor objective, sensorimotor function, psychology and well-being, disability, quality of life, pain and discomfort, and neurotrophic measures.

**CONCLUSION:**

Lack of consensus on outcome measure use hinders comparison of outcomes between nerve injury centers and the development of novel treatments. Development of a core outcome set will help standardize outcome reporting, improve translation of novel treatments from lab to clinical practice, and ensure future research in PNI is more amenable to systematic review and meta-analysis.

**ABBREVIATIONS**
AMED: Allied and Complementary Medicine Database
COS: core outcome set
DASH: Disabilities of the Arm, Shoulder and Hand
MRC: Medical Research Council
NPRS: Numeric Pain Rating Scale
PNI: peripheral nerve injury
PROM: patient-reported outcome measure
RCT: randomized control trial
SF-36: Short Form-36

Peripheral nerve injury (PNI) is a significant health problem, and despite targeted microsurgical interventions, no patient with a major nerve injury ever regains full, preinjury levels of function.^1-4^ This clinical unmet need requires best practices adopted as widely as possible and ultimately new interventions to improve functional outcomes for patients. Several groups worldwide report on a variety of surgical interventions and new discoveries in nerve biology and materials science lend itself to translating new technologies; however, in order to adopt or develop the best of these approaches, clinicians, scientists, and other researchers must be able to compare treatments between patients and between distinct surgical units to allow accurate assessment of their treatment effect. Currently, very few novel treatments are reaching the clinical arena at least in part due to the inconsistent reporting of outcomes following nerve repair surgery.

Multiple outcome measures of nerve regeneration in humans exist, and in previous research these have been inconsistently categorized into several broad domains: sensory, motor, function, patient-reported outcome measures (PROMs), pain, and finally, neurotrophic measures, a term used to describe measures that indirectly examine nerve regeneration at the repair site, end organ, or centrally within the brain. Within each domain several distinct outcome measures exist, but there is no collective agreement across researchers and clinicians in deciding which to use and in what circumstances. Literature reviews have highlighted the inconsistencies in reporting of functional outcomes,^5-7^ and a recent systematic review of outcome reporting in brachial plexus injury has demonstrated the variety in use.^8^ The latter study focused on motor outcome reporting following plexus injury; therefore, the landscape of outcome measure reporting in each of the other critical domains and following PNIs distal to the brachial plexus remains largely unknown.

## Objectives

The aims of this study were to classify outcome measures used in PNI of the upper limb into clinically relevant domains, determine the frequency of use by anatomical site of injury, describe the range of time points after injury where the outcome measures are used, and identify common areas of inconsistencies in their reporting.

## METHODS

### Protocol and Registration

This systematic review is reported in accordance with the Preferred Reporting Items for Systematic Reviews and Meta-Analyses guidelines.^9^ The protocol was registered at the International Prospective Register of Systematic Reviews (PROSPERO), study CRD42018103001, and is available from https://www.crd.york.ac.uk/prospero/display_record.php?ID=CRD42018103001

### Search and Eligibility Criteria

We performed a specific search of the English-language literature on January 8, 2019, using MEDLINE, EMBASE, Allied and Complementary Medicine Database (AMED), and Cochrane Central Register of Controlled Trials databases (all years considered up to the date of the searches). We searched these databases using the following keywords: “peripheral nerve inj*” (key word) or “nerve regeneration” AND “peripheral nerves” (subject headings). The searches were conducted by the principal author, who expanded the key words into corresponding Medical Subject Heading terms. The broad nature of the search was chosen to be as inclusive as possible for potentially relevant studies.

### Inclusion and Exclusion Criteria

**FIGURE 1.**
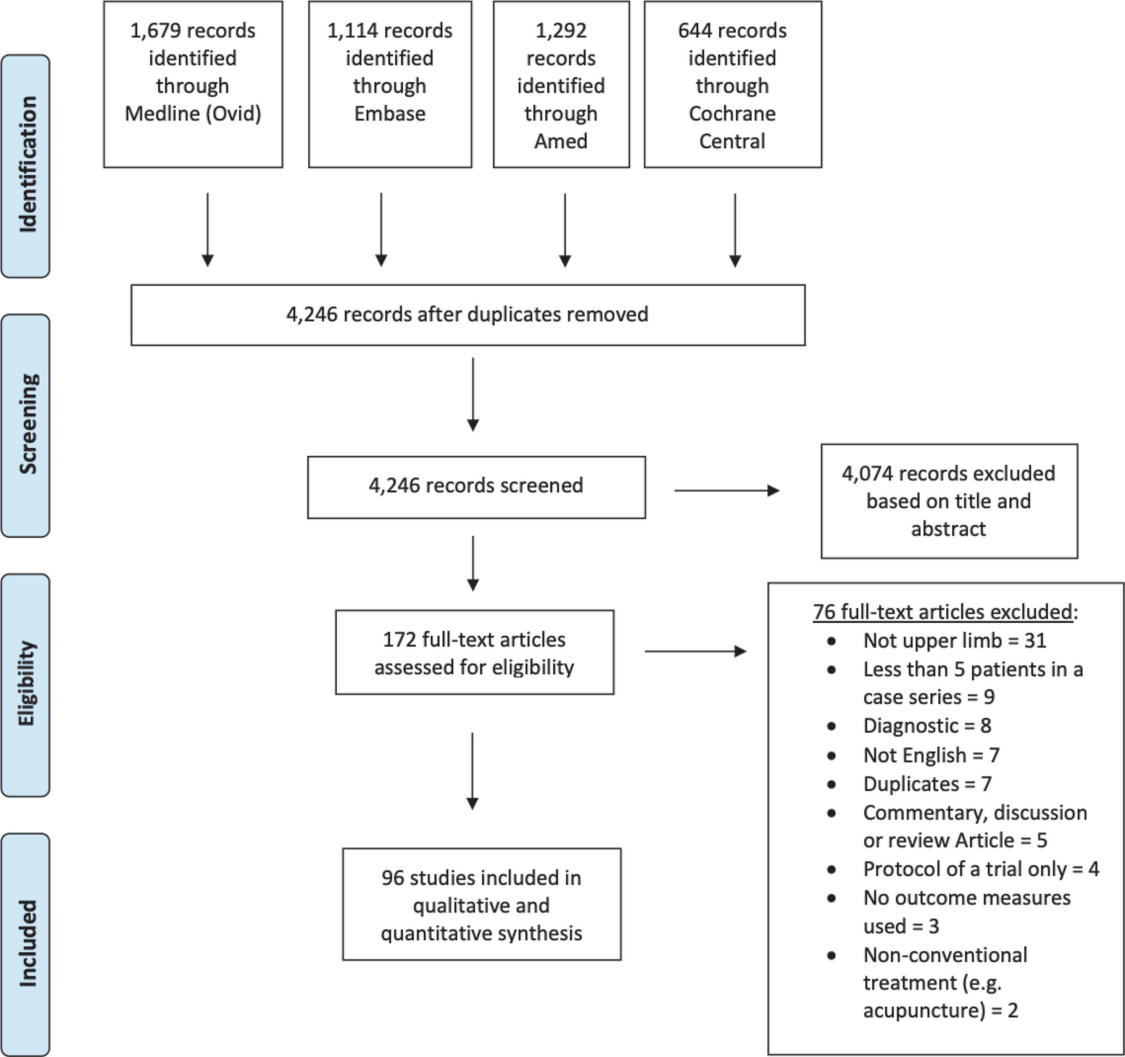
Flowchart demonstrating study selection for inclusion in systematic review.

We included articles that were (1) in English; (2) whose full text was available; and (3) involved adults (>18 yr old) (4) with a PNI (5) of the upper limb, (6) where a measurement of outcome was reported. Exclusion criteria were studies that (1) described cadaveric or non-human studies; (2) were only conference abstracts; (3) did not primarily involve the upper limb (minimum 5 patients with upper-limb injuries); (4) were case series with less than 5 patients; (5) were diagnostic studies; (6) commentaries, discussions, or literature reviews; (7) trial protocols; (8) studies not involving any outcome measures; and (9) those studies employing purely nonconventional treatments for PNI (eg, acupuncture) (Figure 1). Lower limb nerve injuries were excluded, as the functional demands of a lower limb are different and would require a dedicated suite of outcome measures, some of which will overlap with upper limb.

### Study Selection and Data Extraction

All articles underwent a title/abstract screen for inclusion eligibility by 2 independent reviewers (R. M. and S. S.). Those deemed potentially relevant were obtained as full-length papers and screened for eligibility by the same 2 reviewers. After each screening round, the 2 reviewers met to consolidate inclusion/exclusion decisions. Disagreements were arbitrated by the senior author (A. R.).

A data extraction form was produced a priori to collect the following from each article: authors; journal; year of publication; title; geographical location; number of patients; nerves injured; anatomical location of nerves (if available); injury type; intervention; study type; outcome/outcome domain; outcome measurement, eg, technique/instrument; specific metric/format of outcome data from each participant used for analysis and the specific time points used for analysis. Each identified outcome was classified into 1 of 10 outcome domains. Data were extracted independently by 2 reviewers (R. M. and H. E.) and results were consolidated.

### Appraisal and Synthesis of Results

Outcomes were extracted verbatim from source papers and then grouped within a domain based on their given or implied definition using a “best-fit” approach (Table 1).^10^ Sensory and motor domains were further subclassified into objective and subjective subdomains based on the subjective or objective nature of the outcome measure. All authors were involved in this process encompassing a multidisciplinary group of researchers and clinicians. The data extracted from each study using our data extraction proforma was tabulated by domain and accompanied by a narrative review. We calculated how frequently each outcome measure was reported within these domains, how often a named specific instrument was used (eg, scale, clinical test, or piece of equipment) to measure the outcome, how often a specific metric or format of recorded outcome data from each participant was used to record the measurement (eg, force (in g or g/mm^2^)), and the frequency of time-point specification from injury or surgery was recorded.

**TABLE 1.** Outcome Measure Domains

Outcome measure domain	Definition	Examples
Sensory objective	Objective assessment of sensory receptor reinnervation	Tactile gnosis (static 2-point discrimination)
Sensory subjective	Subjective assessment of sensory receptor reinnervation	Medical Research Council Sensory Scale
Motor objective	Objective assessment of muscle reinnervation	Dynamometry of grip or pinch strength
Motor subjective	Subjective assessment of muscle reinnervation	Muscle strength (British Medical Research Council Grading)
Sensorimotor function	Objective assessment of composite functions (combined sensory and motor reinnervation)	Moberg's pickup test
Psychology and well-being	Assessment of the psychological progress during regeneration	Hospital Anxiety and Depression Scale
Disability	Assessment of disability caused by peripheral nerve injury	Disabilities of the Arm, Shoulder and Hand (DASH) score; Groningen Activity Restriction Scale
Quality of life	Assessment of quality of life after peripheral nerve injury	The sense of coherence 13-item scale
Pain and discomfort	Assessment of the pain or discomfort felt by the patient after peripheral nerve injury	The Numeric Pain Rating Scale
Neurotrophic measures (end organ) (at the repair site) (central nervous system [CNS])	Assessment of regeneration along the anatomical axis of the peripheral nervous system after injury	End organ: computed tomography cross-sectional area of muscle Repair site: Tinel's test CNS: functional magnetic resonance imaging

We subsequently grouped outcome measures by anatomical site of injury in order to identify the most commonly used (in 3 or more studies) outcome measures for hand sensory nerves, mixed (motor and sensory) upper limb nerves, and nerves of the brachial plexus.

## RESULTS

### Study Selection

The electronic database search yielded 4246 articles, of which 96 remained eligible for inclusion (Figure 1). Of the 96 studies included in the final analysis, there were 15 randomized control trials (RCTs), 8 case-control studies, 18 cohort studies, 5 observational studies, and the remainder were case-series or retrospective reviews. A total of 56 individual outcome measures were utilized across 28 different countries with 7097 patients included. A total of 16 studies involved injury to the brachial plexus, 59 studies involved injury to mixed (motor/sensory) upper limb nerves (distal to the brachial plexus and proximal to the hand), and 17 studies involved sensory nerve injuries of the hand.

### Domain Categorization

Ten domains were used to categorize the 56 outcome measures identified through our search (Table 1). No study included all 56 outcome measures nor did any study utilize an outcome measure from all 10 domains. The most widely utilized outcome measures were 2-point discrimination, static (30 studies) or moving (16 studies) to assess tactile discrimination and the Medical Research Council (MRC) Scale for assessment of motor function (30 studies).

A more detailed review of outcome measures used within each domain is included in **Supplementary Tables 1-8**.

### Specific Instrument

Only one study did not specify the instrument being used for quantification of results. Gordh et al^11^ used a cold metal roller to assess cold at baseline but did not perform any further measurements and did not specify the method of quantitation of the cold threshold. Sensory and motor outcomes (**Supplementary Tables 1** and **2**) were most often assessed using well-known instruments. Sensorimotor function and psychology and well-being (**Supplementary Tables 3** and **4**) were frequently assessed by a range of different instruments or scoring systems with no commonly (used in 3 or more studies) used instruments. Disability (**Supplementary Table 5**) was most commonly assessed using the Disabilities of the Arm, Shoulder and Hand (DASH) PROM and was used in 8/11 studies assessing disability. Quality of life (**Supplementary Table 6**) was commonly assessed using the Short Form-36 (SF-36) PROM and was used in 4/7 studies assessing quality-of-life outcomes. Pain and discomfort was measured using a variety of instruments (**Supplementary Table 7**) but pain intensity scales (visual analog scale [VAS] and Numeric Pain Rating Scale [NPRS]) were the most commonly used in 17/18 studies assessing pain and discomfort. Electrophysiology was the most commonly used instrument to assess neurotrophic outcomes (**Supplementary Table 8**) in 14/17 studies.

### Specific Metric Usage

The specific metric or unit of outcome measurement was uniformly well described in all domains (**Supplementary Tables 1-8**).

### Time Points for Assessment

**FIGURE 2.**
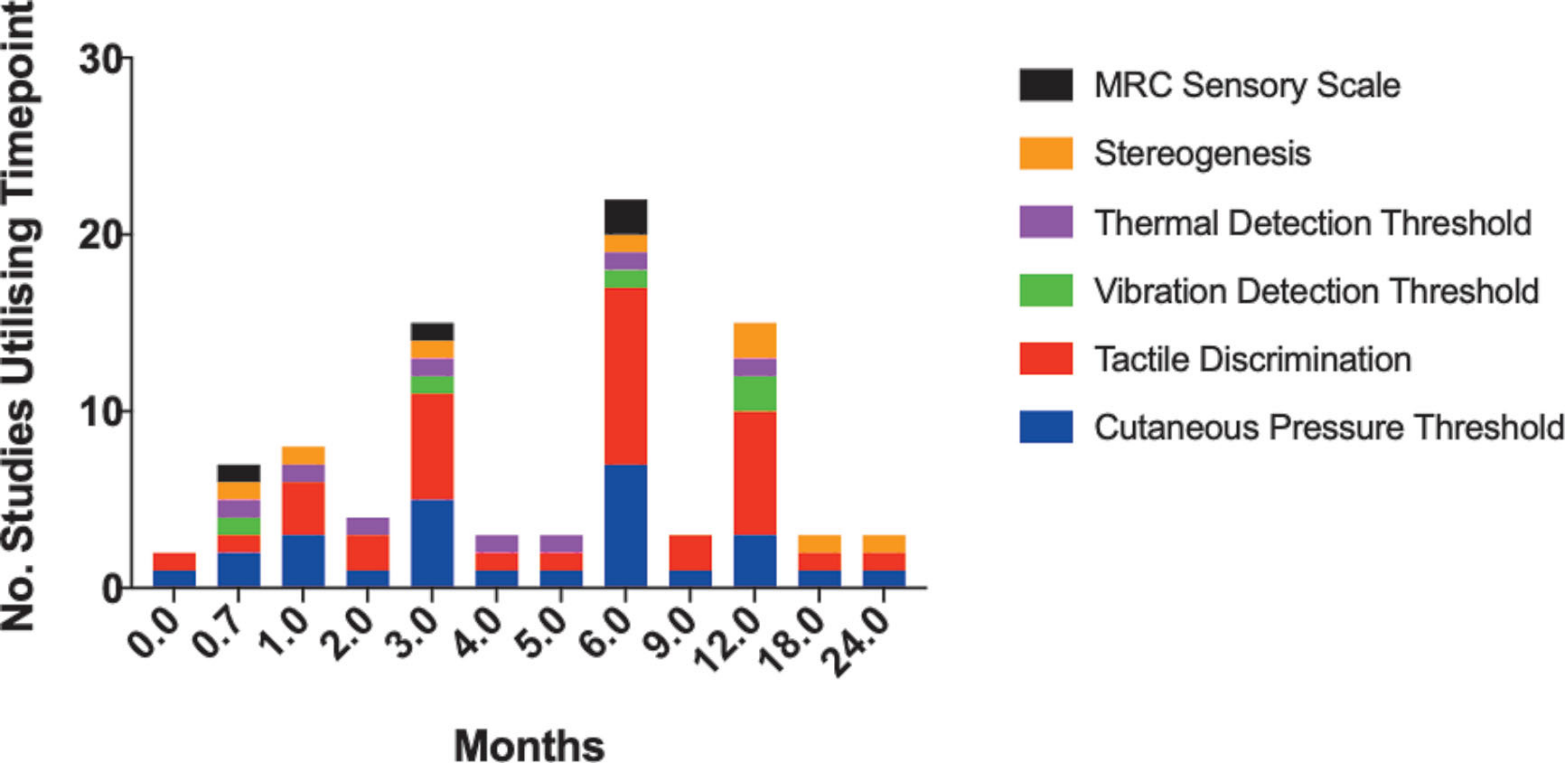
Cumulative bar chart of time points for sensory outcome measure use. Time points for the usage of sensory outcome measures varied widely; however, there was a clear trend in their use. Most studies obtained a baseline or early estimate of sensation at time 0, 3 wk, or 1 mo after surgery (or after injury in observational studies). After this, the majority of studies utilized sensory outcome measures at 3 and 6 mo with the final assessment at 12 mo. A small minority of studies continued sensory assessments of any modality past 12 mo, with no study making a sensory assessment after 24 mo.

**FIGURE 3.**
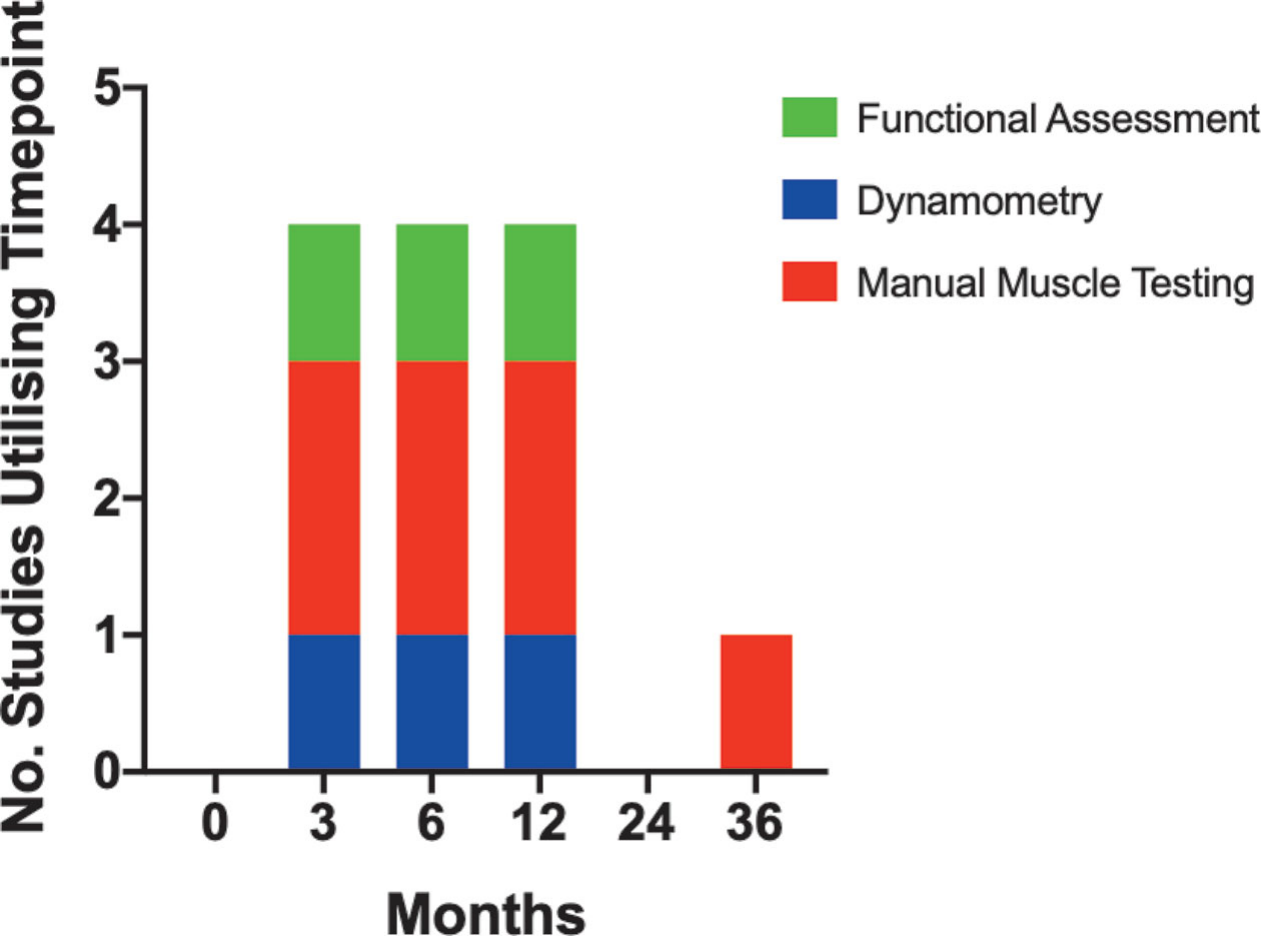
Cumulative bar chart of time points for motor outcome measure use. Time points for the usage of motor outcome measures followed a common trend. No study undertook a baseline assessment up to 1 mo after surgery or injury. Instead, all studies measured outcomes at 3, 6, and 12 mo. Similarly, to sensory outcomes, the end point for motor outcome assessment was 12 mo with only 1 study making an assessment after this at 36 mo.

The time points from injury or surgery used for assessment of outcomes were highly variable with the majority of studies defining a range as opposed to specific time points. For sensory and motor outcome measurements, most studies specified time points for assessment since injury/surgery and thus a more detailed analysis was performed (Figures 2 and 3). Most studies performed sensory and motor assessments at 3, 6, and 12 mo after injury/surgery, with only 4 studies continuing sensory or motor measurements after 12 mo of follow-up.

Disability outcome reporting time points were specified in 3/8 studies (**Supplementary Table 5**) utilizing the DASH which was used monthly post-injury/surgery up to 6 mo in hand sensory nerve injured patients^12^; at 3, 6, and 12 mo postoperatively in mixed (sensory/motor) upper limb nerve injuries^13^ and at 12 mo postoperatively in patients undergoing nerve transfers for upper root brachial plexus injuries.^14^ The SF-36 PROMs’ usage time points for the assessment of quality of life were specified in 2/4 studies, at baseline, and then at 6 mo post-surgery^15^ and at 7 time points over a 15-wk period in a drug trial to treat neuropathic pain.^11^ The time points for the use of pain intensity scales VAS and NPRS since PNI or surgery was often specified (10/12 studies utilizing VAS and 5/5 studies utilizing NPRS). VAS was used preoperatively, monthly in the first 6 mo, then every 6 mo for 2 yr^16^ and preop then 1, 3, and 6 mo after surgery.^15^ The NPRS was used 1 wk preop and 1, 3, and 6 mo postoperatively.^17^ Neurotrophic measures were used at wide-ranging time points after injury/surgery (**Supplementary Table 8**) with only 4/17 studies specifying time points for assessment of which 3 utilized electrophysiology. This was used at 1, 3, 6, 12, 18, and 24 mo^18^; 8 and 12 mo^19^; and 12, 18, and 24 mo postoperatively.^20^

### Summary of Outcome Measure Use at Different Anatomical Sites of Injury

#### Brachial Plexus (Total = 16 Studies)

**FIGURE 4.**
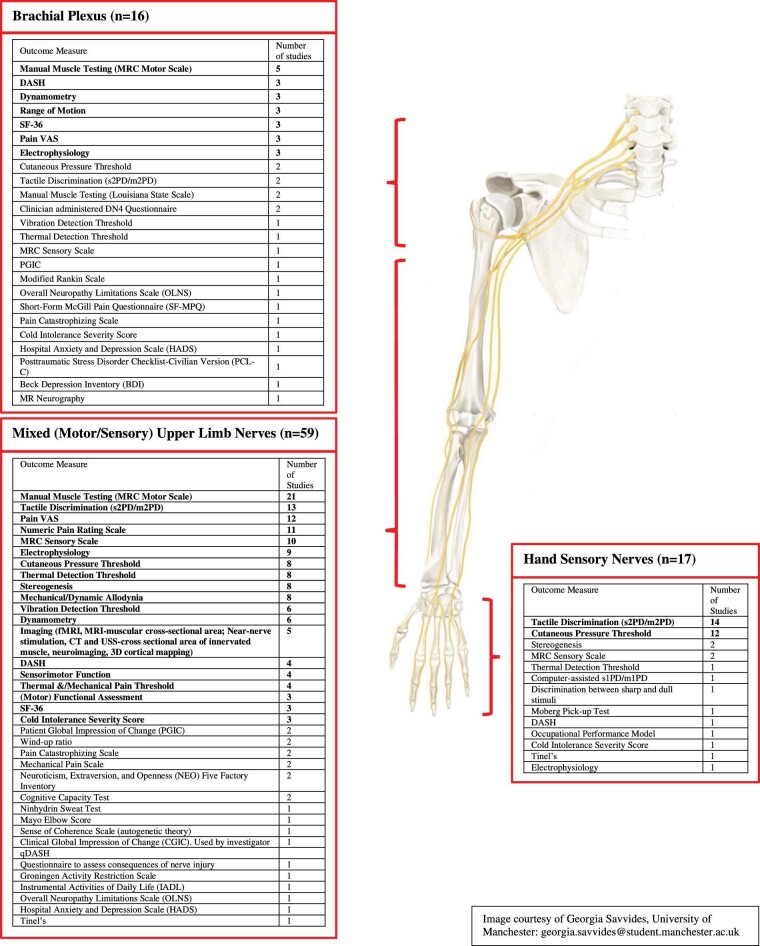
Frequency of outcome measure use by anatomical location of peripheral nerve injury (brachial plexus, mixed (motor/sensory) upper limb nerves, purely sensory nerves of the hand) (4 studies did not specify anatomical location of nerve injuries).

In 16 studies of brachial plexus injures, outcome measures were reported across 6 domains with a clear focus on motor assessment: motor objective (3/16 studies) and motor subjective (8/16 studies), disability (DASH, 3/16 studies), quality of life (SF-36, 3/16 studies), pain and discomfort (VAS, 3/16 studies), and neurotrophic measures (electrophysiology, 3/16 studies) (Figure 4).

#### Mixed (Motor and Sensory) Upper Limb Nerve Injury (Total = 59 Studies)

In mixed (motor and sensory) upper limb (distal to brachial plexus and proximal to wrist) nerve injury studies, there was a much broader spread of outcome measure use with 41 outcome measures used across 59 studies (Figure 4). Subjective motor assessment, the MRC Motor Scale, was the most commonly used single outcome measure (21/59 studies). However, sensory outcome measures were chosen in 54/59 studies compared to motor outcome measures in only 32/59 studies. Pain and discomfort (44/59 studies) most commonly assessed using VAS (12/59 studies) or NPRS (11/59 studies). Novel neurotrophic measures were utilized in 5/59 studies. Studies examined the central nervous system response to PNI using functional magnetic resonance imaging (MRI), 3D cortical mapping/thickness (MRI), or cerebral blood flow (positron emission tomography scanning) or they examined end-organ changes, most commonly muscle cross-sectional area using either MRI, computerized tomography, or ultrasound scanning (**Supplementary Table 8**).

#### Hand Sensory Nerve Injury (Total = 17 Studies)

In 17 studies of hand sensory nerve injuries, objective sensory measures were most commonly utilized with tactile discrimination (14/17 studies) and cutaneous pressure threshold (12/17 studies), the 2 most commonly used (Figure 4). Only 2/17 studies assessed disability after hand sensory nerve injury with one study using the DASH questionnaire and another using the occupational performance model.^21^

## DISCUSSION

This systematic review highlights inconsistencies in use and reporting of outcome measures and their results within the PNI literature. This is important because a lack of clarity and standardization of assessment in these life changing injuries precludes meaningful comparisons between patients and across patient cohorts subject to differing interventions. Furthermore, this has a negative impact on the translation of novel treatments or outcome methodologies into clinical trials. To date, there have been no systematic reviews of outcome measure use across the large and varied spectrum of clinical upper limb PNI research.

Systematic reviews of outcome measure use in clinical brachial plexus research have demonstrated a focus on measures of motor recovery with a lack of data collected on patient-centered outcomes such as quality of life or the effect on mental health, specifically anxiety, and depression.^8,22^ We have found similar results in our systematic review, which has highlighted a clear trend toward motor outcome reporting in brachial plexus studies, with a focus on subjective measures versus objective measures of motor function (**Supplementary Table 2** and Figure 4). This may be due to a lack of PROMs available for use after PNI that effectively assess patient symptoms and function.^23^ In our systematic review, we have also demonstrated a widely heterogeneous use of outcome measures in mixed upper limb nerve injuries and a clear focus on objective sensory outcome measures in hand sensory nerve injury with only 2 studies assessing disability, using tools that are not validated for use in hand sensory nerve injury.

Time points for assessment were highly variable and appeared to follow arbitrary end points. In distal sensory injuries or mixed upper limb nerve injuries where the regenerative distance to the end organ is less than 300 mm, follow-up of 12 mo is logical given regenerative rates of approximately 1 mm/day in humans.^24,25^ In brachial plexus injuries where the distance to the end organ can be 800 mm or more,^25^ follow-up of 2 to 3 yr may be more appropriate. Standardizing time points for measurement will ensure comparable phases of post-treatment recovery, which is particularly important for PNI at different levels of injury.

Our primary aim was to describe and classify outcome measures used in clinical PNI research. The 56 individual outcome measures were grouped into 10 core domains based on their implied definition and using a “best-fit” approach. There is no consensus on how to categorize outcome domains^26-28^ and where domain taxonomies have been published for medical research^27^ it was unclear how PNI outcome measures would fit into these domains. Previous reviews have suggested domains such as sensory function, motor function, pain and discomfort, PROMs, and neurophysiological outcome measurements.^6,7^ We utilized these domains within our taxonomy but added new domains given the large number of diverse PNI outcome measures identified.

Within this domain structure, each outcome measure is likely to be of differing importance to the critical stakeholders (patients/clinicians/researchers/healthcare funders); however, per-haps the most significant determinant of their relevance will be the anatomical level of PNI. Therefore, allocating outcome measures within each domain that are appropriate to anatomical level seems a pragmatic next step. Historically, there has been a focus on domains such as sensory and motor function as a barometer of success of repair and regeneration. While biological outcomes are clearly important, PNI and its complex interplay with the brain, highlighted eloquently in Lundborg's “The Hand and the Brain,”^29^ cannot so easily be assessed using just biological outcome measures. Domains such as psychology and well-being, disability, and quality of life need to be explored further and included in future assessment of PNI patients. In addition, neurotrophic measures utilizing the latest imaging technology are likely to provide even greater insight into the interplay between PNI and the central nervous system.

Collating and describing these measures into domains allows us to begin the process of stratifying this wide selection of outcome measures into a core outcome set (COS). The Core Outcome Measures in Effectiveness Trials initiative began in 2010 to develop COS in clinical trials.^10^ They have published a set of 11 standards required for the development of COS.^30^ This systematic review has met the first 4 of these standards that look to specify the scope of the COS in PNI surgery. The remaining standards require definition of the stakeholders involved (standards 5-7) and subsequently development of the consensus process (standards 8-11) utilizing the scope of the COS between stakeholders.^30^

### Limitations

Limitations of this study include the search for articles that were solely retrieved from established databases with no additional manual searches conducted; in addition, articles not published in English were excluded. International publications were included, however. The results of this systematic review reveal important trends in outcome measure use in PNI research that could be used to help inform the development of a core outcome set.

## CONCLUSION

We have described and categorized outcome measure use in clinical upper limb PNI research identifying a lack of consensus among researchers over which outcome measures to use for a particular PNI. Common time points for the use of sensory and motor outcome assessment have been established, and we have demonstrated a lack of validated measures of psychology and well-being, disability, and quality of life after PNI of the upper limb.

We now need to develop a COS of validated outcome measures for PNI research that are inclusive of patient-reported measurements of psychology and well-being, disability, and quality of life. Objective and sensitive measures of PNI that are inclusive of all stakeholders (patients, clinicians, and researchers) will allow us to collect effective data that are comparable between PNI centers both nationally and internationally. In addition, it would allow researchers and policymakers to develop more accurate guidelines for the management of PNI patients, which in turn would standardize care and improve outcomes.

### Funding

The authors acknowledge support from the Engineering and Physical Sciences Research Council (EPSRC) and the Medical Research Council (MRC) Centre for Doctoral Training (CDT) in Regenerative Medicine (EP/L014904/1), and the Royal College of Surgeons/Sir John Fisher Foundation Research Fellowship.

### Disclosures

The authors have no personal, financial, or institutional interest in any of the drugs, materials, or devices described in this article.

## Supplementary Material

nyab060_Supplemental_Files

## Notes

**Supplementary Table 1**. Sensory outcome reporting.

**Supplementary Table 2**. Motor outcome reporting.

**Supplementary Table 3**. Sensorimotor function outcome reporting.

**Supplementary Table 4.** Psychology and well-being outcome reporting.

**Supplementary Table 5.** Disability outcome reporting.

**Supplementary Table 6.** Quality of life outcome reporting.

**Supplementary Table 7.** Pain and discomfort outcome reporting.

**Supplementary Table 8.** Neurotrophic measure outcome reporting.

## COMMENTS

Systematic analysis of outcome measures used in PNI is a very important topic. To date the focus is on maximum obtainable motor power of single muscles, not on function per se. With some gap this is followed by grading of sensory deficit and pain.

The authors deliver a concise analysis that is arranged according to anatomy. A considerable amount of data was worked-up, and is presented in a table and graphic format that helps to extract specific information. The endeavor is very worthwhile, and demonstrates and documents the current state of grading deficit after UE nerve injury and outcome following repair. The logical next will be an analysis for the LE.

A much needed next step will be to re-evaluate if the used outcome measures are sufficient to report on- and compare functional aspects (compare proposals by Dave Kline, grading upper and lower extremity nerve injuries in Nerve Injuries-Operative Results for major nerve injuries, entrapments, tumors 1995 1st Ed; or the Raimondi/Gilbert grading systems for OBPP). Description of gained function eg, active movement is much helped by video documentation and analysis. Sports science and performance specialists have an expertise and since long use systematic video analysis. Graphic evaluation programs are commercially available. Interpretation still is a matter of expertise and very individual (= not systematic). However, due to costs, time and effort comparable video analysis (not video documentation) so far did not find its way in clinical practice of care for PNI patients.

So new efforts in that direction will be more than welcome, and papers like this might be a starting point. Congratulation!

**Thomas Kretschmer**

*Oldenburg, Germany*

In this work, the authors have systematically reviewed clinical outcome measure use in upper limb peripheral nerve injuries literature. The authors should be commended for their endeavor stretching over a wide variety of surgeons, care givers and institutions with over 7000 international patients in this quantitively challenging analysis. Their work reveals the inconsistency of what and how measures are reported across centers and highlights the lack of uniform standards.

While subjective and objective components of patient evaluation, in both physical, mental as well as disabling pain and quality of life factors will always remain a hot topic of controversary in their interpretation and validity, we are left with limited patient consistent report tools. Furthermore, significant variations in multiple fields over time across the collected data of this review along with lack of standardization are well-known inherent limitations which, still unfortunately, significantly limit future conclusions. Still, we support the authors opinion and need for a future multidisciplinary consensus process addressing prioritization and stratification of measurable and less-measurable factors in both upper and lower extremity injuries. In particular, the authors attempt to develop a unified and verified future outcome set of a ten-domain core set of measures, incorporating objective, as well as subjective, measurements may serve as an actionable avenue. We await future reports from this group pursuing their call-for-action across the nerve community.

**Daniel Umansky**

**Rajiv Midha**

*Calgary, Alberta*
