# Supplementary material for: A Quantitative Systematic Review of Clinical Outcome Measure Use in Peripheral Nerve Injury of the Upper Limb
Source: Neurosurgery. 2021 Mar 8;89(1):22–30. doi: 10.1093/neuros/nyab060 (PMC8203424; doi:10.1093/neuros/nyab060)
Supplement: nyab060_Supplemental_Files [file nyab060_supplemental_files.zip › SR Outcome Measures PNI.Supplementary Table 1.docx]

Supplementary Table 1: Sensory outcome reporting

| Outcome Measure Domains | Outcome Measures | No. of studies reporting outcome measure | Instrument | Metric | Specific Time points |
| --- | --- | --- | --- | --- | --- |
|  |  |  |  |  |  |
| Sensory  Objective | Cutaneous Pressure Threshold | 25 | 25 | 23 | 8 |
|  | Static Tactile  Discrimination (2PD) | 30 | 30 | 28 | 13 |
|  | Moving Tactile Discrimination (2PD) | 16 | 16 | 16 | 5 |
|  | Vibration Detection Threshold | 7 | 7 | 7 | 2 |
|  | Thermal Detection Threshold | 10 | 9 | 9 | 5 |
|  | Stereogenesis | 10 | 10 | 8 | 3 |
|  | Mechanical detection threshold | 4 | 4 | 4 | 2 |
|  | Ninhydrin sweat test | 1 | 1 | 1 | 0 |
| Sensory  Subjective | MRC Sensory  Scale | 16 | ̶̶ | 16 | 2 |
|  | Discrimination between sharp and dull stimuli | 1 | 1 | 1 | 0 |

*Sensory*

*Sensory Objective*

Eight specific outcomes measures were classified under the sensory outcome domain. These were further subdivided into objective (9 outcome measures, used a total of 94 times) and subjective measures (2 outcome measures, used a total of 17 times) with a clear tendency towards the use of objective measures. The two most commonly assessed sensory objective outcomes were tactile discrimination and cutaneous pressure threshold.

Tactile discrimination, or the ability to discriminate between two points using touch alone, was utilised in a total of 31 studies. Of these 31 studies, it was assessed using static two-point discrimination (2-PD) in 30 studies ^1–30^ and/or moving 2-PD in 16 studies ^1,3–5,7–9,14,15,18,24,28–32^. It was uniformly measured in millimetres but was occasionally ^13,28,30^ additionally stratified using the Mackinnon/Dellon modified Highet Classification ^33^ which groups scores according to the Highet Classification but with additional s2PD and m2PD measurements. Others stratified the raw measurements using the Rosen Score ^34^, Dellon’s Highet Scale ^33^ or the American Society for Surgery of the Hand classification (as described by Moberg)^35^. The time points for assessment varied widely between studies. If specified, in digital/hand sensory nerve injury studies the range of follow up times varied from 3 weeks up to 87 months after surgery with the most common time points for assessment at 6 and/or 12 months (8 studies). In mixed upper limb nerve injuries, the range of recorded follow up times was between 1 month and 8 years after injury, with the most common period for follow up being between 12 – 24 months after injury (6 studies). After brachial plexus injury tactile discrimination was most commonly assessed between 2 – 4 years after surgery (2 studies).

Cutaneous pressure threshold assessed using Semmes-Weinstein Monofilaments ^36^ or von Frey filaments ^35^ was utilised in a total of 25 studies ^1,2,16,17,19,21,24–26,28,30,31,3,32,37–40,4–7,10,11,13^ all of which specified the instrument used. The specific metric used for reporting was force (in g or g/mm^2^) in 8 studies or score (1-5) based on individual monofilaments where each monofilament is given a score instead of its actual force ^36^ in 7 studies. Colour of the monofilament was used in 3 studies, whilst the remaining studies used: the fibre size or a visual analogue scale. The time points for assessment in digital/hand sensory nerve injury studies varied but were most commonly found between 6 – 24 months after surgery. In mixed upper limb nerve injuries, the range of recorded follow up times was between 1 month and 10 years after injury although most studies made assessments between 6 – 24 months. After brachial plexus surgery time points for assessment ranged between 1 – 5 years.

The vibration detection threshold was assessed in 7 studies ^4,7,22,23,37,41,42^ using either a tuning fork (256Hz) or a bio-thesiometer. A bio-thesiometer is a handheld mains operated rubber tactor (antenna) which vibrates at 100 Hz displaying a linear scale showing the applied voltage where an increase in voltage increases the amplitude of vibration ^43^. All studies described a specific metric in reporting outcomes using binary terms of perceptible (+) or not (-) (Foroni et al. ^4^) or the specific threshold limit voltage (Taylor et al. ^37^). Lundborg et al. ^7^ used their own previously described sensibility index ^44^. Six studies assessing mixed upper limb nerve injuries assessed outcomes at a range of time periods from 3 weeks up to 8 years after surgery but where specified these were assessed at 3, 6 and 12 months ^7^ and 3 weeks and 1 year after surgery ^23^. One brachial plexus study assessed outcomes at a mean of 41 months (range 36-52 months) ^4^.

The thermal detection threshold was utilised in 10 studies ^4,13,22,23,40–42,45–47^. Wong et al. ^13^ assessed the cold detection threshold using just noticeable differences ^48^ (which is the minimal difference required to detect between two stimuli of close intensity) on a monthly basis between 1-6 months after digital nerve injury. Foroni et al. ^4^ assessed temperature perception in a cohort of pan-plexus injured patients treated with nerve transfers (specifically investigating intercostobrachial nerve (ICBN) as a donor of sensory fibres to the lateral cord contribution to the median nerve (LCMN)) using a steel bar warmed to 50°C and an ice bar recorded as perceptible (+) or not (-). Time points for assessment were between 36-52 months with a mean of 41 months post-operatively. Taylor et al., Goswami et al. and Vollert et al. ^22,23,41^ used a computer-controlled (Peltier- (TSA-II NeuroSensory Analyzer, Medoc Ltd., Israel), whilst Gottrup et al., Attal et al. and Witting et al. ^40,45,46^ used a thermotester (SOMEDIC,Hörby, Sweden)) device with thermal probe to assess cold and warm detection threshold respectively, using a method of limits, whereby the stimulus intensity increases linearly or exponentially from a neutral temperature until the subject stops it immediately as they detect the specified sensation ^48^. Time points for assessment in these studies varied considerably with no commonly used assessment period post-surgery.

Stereogenesis, or the ability to detect three-dimensional objects via touch, was most commonly (5/10 studies) ^2,10,22,23,25^ assessed using the shape-texture identification test described by Rosen and Lundborg ^49^. Two studies ^28,29^ utilised an object identification test originally described by Dellon ^50^ and one study ^19^ utilised the grating orientation test ^51^ which consists of gratings of parallel bars and grooves of equal widths on hemispherical plastic domes. Subjects are required to touch the domes and indicate which direction the grooves and bars are before the stimulus is removed. Bertelli et al. ^38^ undertook a locognosia assessment using a 2.0 Semmes Weinstein monofilament and Adson forceps in order to localise touch, whereas Hsu et al. ^3^ utilised a manual tactile test to assess stereogenesis and barogenesis. The Rosen Score was the most commonly used metric for reporting results (3/10 studies), whereas those studies using Dellon’s object identification test reported time and correct number/percentage of objects identified. Time points for assessment in digital sensory nerve injury studies were at a minimum of 12 months after surgery up to 87 months, whereas in mixed nerve injury studies assessment timepoints ranged from 1 month after surgery up to 8 years. The most common time points for assessment were between 18 months and 3 years after surgery.

The mechanical detection threshold was assessed in 4 studies ^9,23,41,42^ using either von Frey hairs with rounded tips (to avoid nociceptor activation) between 0.25 and 512 mN or a pressure-specifying sensory device ^9^. Goswami et al. ^23^ specified time points for assessments at 2-3 weeks after surgery and after one year post-mixed upper limb nerve injury when using the mechanical detection threshold, whereas Bertleff et al. ^9^ assessed hand sensory nerve injury patients repaired using a nerve conduit at 3, 6 and 12 months.

Walton et al. ^52^ was the only author to use the Ninhydrin sweat test to assess sudomotor nerve regeneration after nerve grafting for mixed upper limb nerve injury. A positive or negative result was recorded, and it was utilised between 10-31 months after surgery.

*Sensory Subjective*

Subjective sensory outcome measures were utilised far less than objective measures. The original Highet ^53^ classification (S0-4) of sensory recovery, devised during the second world war, was used in 5 studies ^54–58^ and often termed the MRC sensory scale, despite being a misnomer ^59^. The modification of the original Highet classification by Mackinnon-Dellon (1988) ^33^ which includes further 2-PD criteria was used more often ^5,26,52,54,60–65^. Other modifications in the 20^th^ Century include the Millesi score ^59^ which combines ratings of joint motion in the hand, 2-PD and a pick-up test in addition to strength measurements which was utilised by one group ^66^. Where specified Cheng et al. ^5^ used the modified Highet classification in hand sensory nerve injured patients at 3 weeks and 6 months post op. Bai et al. ^58^ used the original Highet classification in mixed upper limb nerve injuries at 3, 6 and 12 months post-operatively.

References

1. Cheng A. Use of early tactile stimulation in rehabilitation of digital nerve injuries. *Am J Occup Ther*. 2000;54(2):159-165.

2. Krarup C, Rosen B, Boeckstyns M, Sorensen A, Lundborg G, Moldovan M. Sensation, mechanoreceptor, and nerve fiber function after nerve regeneration. *Ann Neurol*. 2017;82(6):940-950. doi:http://dx.doi.org/10.1002/ana.25102

3. Hsu H, Shieh S, Kuan T, et al. Manual tactile test predicts sensorimotor control capability of hands for patients with peripheral nerve injury. *Arch Phys Med Rehabil*. 2016;97(6):983-990.

4. Foroni L, Siqueira MG, Martins RS, Heise CO, Sterman HN, Imamura AY. Good sensory recovery of the hand in brachial plexus surgery using the intercostobrachial nerve as the donor. *Arq Neuropsiquiatr*. 2017;75(11):796-800. doi:https://dx.doi.org/10.1590/0004-282X20170148

5. Cheng AS, Hung L, Wong JM, et al. A prospective study of early tactile stimulation after digital nerve repair. *Clin Orthop Relat Res*. 2001;(384):169-175. http://ovidsp.ovid.com/ovidweb.cgi?T=JS&PAGE=reference&D=med4&NEWS=N&AN=11249162

6. Saeki M, Tanaka K, Imatani J, et al. Efficacy and safety of novel collagen conduits filled with collagen filaments to treat patients with peripheral nerve injury: a multicenter, controlled, open-label clinical trial. *Injury*. 2018;49(4):766‐774. doi:10.1016/j.injury.2018.03.011

7. Lundborg G, Rosén B, Dahlin L, Danielsen N, Holmberg J. Tubular versus conventional repair of median and ulnar nerves in the human forearm: early results from a prospective, randomized, clinical study. *J Hand Surg Am*. 1997;22(1 CC-Child Health CC-Bone, Joint and Muscle Trauma CC-Neuromuscular):99‐106. doi:10.1016/S0363-5023(05)80188-1

8. Rinker B, Liau JY. A prospective randomized study comparing woven polyglycolic acid and autogenous vein conduits for reconstruction of digital nerve gaps. *J Hand Surg Am*. 2011;36(5 CC-Neuromuscular):775‐781. doi:10.1016/j.jhsa.2011.01.030

9. Bertleff MJOE, Meek MF, Nicolai JPA. A prospective clinical evaluation of biodegradable Neurolac nerve guides for sensory nerve repair in the hand. *J Hand Surg Am*. 2005;30(3):513-518. doi:10.1016/j.jhsa.2004.12.009

10. Rosén B, Björkman A, Lundborg G. Improved sensory relearning after nerve repair induced by selective temporary anaesthesia - a new concept in hand rehabilitation. *J Hand Surg Br*. 2006;31(2 CC-Anaesthesia):126‐132. doi:10.1016/j.jhsb.2005.10.017

11. He B, Zhu Q, Chai Y, et al. Safety and efficacy evaluation of a human acellular nerve graft as a digital nerve scaffold: A prospective, multicentre controlled clinical trial. *J Tissue Eng Regen Med*. 2015;9(3):286-295. doi:10.1002/term.1707

12. Sungpet A, Suphachatwong C, Kawinwonggowit V. One-fascicle median nerve transfer to biceps muscle in C5 and C6 root avulsions of brachial plexus injury. *Microsurgery*. 2003;23(1):10-13. http://ovidsp.ovid.com/ovidweb.cgi?T=JS&PAGE=reference&D=med4&NEWS=N&AN=12616512

13. Wong JN, Olson JL, Morhart MJ, Chan KM. Electrical stimulation enhances sensory recovery: a randomized controlled trial. *Ann Neurol*. 2015;77(6):996‐1006. doi:10.1002/ana.24397

14. Battiston B, Tos P, Cushway TR, Geuna S. Nerve repair by means of vein filled with muscle grafts I. Clinical results. *Microsurgery*. 2000;20(1):32-36. doi:http://dx.doi.org/10.1002/%28SICI%291098-2752%282000%2920:1%3C32::AID-MICR6%3E3.0.CO;2-D

15. Mailander P, Berger A, Schaller E, et al. Results of primary nerve repair in the upper extremity. *Microsurgery*. 1989;10(2):147-150. http://ovidsp.ovid.com/ovidweb.cgi?T=JS&PAGE=reference&D=emed4&NEWS=N&AN=19181477

16. Bjorkman A, Rosen B, Lundborg G. Enhanced function in nerve-injured hands after contralateral deafferentation. *Neuroreport*. 2005;16(5):517-519. doi:http://dx.doi.org/10.1097/00001756-200504040-00020

17. Martins RS, Siqueira MG, Heise CO, Foroni L, Teixeira MJ. A prospective study comparing single and double fascicular transfer to restore elbow flexion after brachial plexus injury. *Neurosurgery*. 2013;72(5):709-715. doi:https://dx.doi.org/10.1227/NEU.0b013e318285c3f6

18. Weber RA, Breidenbach WC, Brown RE, Jabaley ME, Mass DP. A Randomized Prospective Study of Polyglycolic Acid Conduits for Digital Nerve Reconstruction in Humans. *Plast Reconstr Surg*. 2000;106(5):1036-1045. doi:10.1097/00006534-200010000-00013

19. Klein HJ, Fakin RM, Ducommun P, et al. Evaluation of Cutaneous Spatial Resolution and Pressure Threshold Secondary to Digital Nerve Repair. *Plast Reconstr Surg*. 2016;137(4):1203-1212. doi:http://dx.doi.org/10.1097/PRS.0000000000002023

20. Chow SP, Luk DK, Ngai YY, Hwang JC. Immediate return of sensation after digital nerve repair. *Aust N Z J Surg*. 1980;50(3):228-232. http://ovidsp.ovid.com/ovidweb.cgi?T=JS&PAGE=reference&D=med2&NEWS=N&AN=6931580

21. Nunley JA, Ugino MR, Goldner RD, Regan N, Urbaniak JR. Use of the anterior branch of the medial antebrachial cutaneous nerve as a graft for the repair of defects of the digital nerve. *J Bone Joint Surg Am*. 1989;71(4):563-567. http://ovidsp.ovid.com/ovidweb.cgi?T=JS&PAGE=reference&D=med3&NEWS=N&AN=2703516

22. Taylor KS, Anastakis DJ, Davis KD, et al. Chronic pain and sensorimotor deficits following peripheral nerve injury. *Pain*. 2010;151(3):582-591. doi:http://dx.doi.org/10.1016/j.pain.2010.06.032

23. Goswami R, Anastakis DJ, Katz J, Davis KD. A longitudinal study of pain, personality, and brain plasticity following peripheral nerve injury. *Pain*. 2016;157(3):729-739. doi:http://dx.doi.org/10.1097/j.pain.0000000000000430

24. Aszmann OC, Muse V, Dellon AL. Evidence in support of collateral sprouting after sensory nerve resection. *Ann Plast Surg*. 1996;37(5):520-525. http://ovidsp.ovid.com/ovidweb.cgi?T=JS&PAGE=reference&D=med4&NEWS=N&AN=8937606

25. MahmoudAliloo M, Bakhshipour A, Hashemi T, AR R, Hassan-Zadeh R. The correlation of cognitive capacity with recovery of hand sensibility after peripheral nerve injury of upper extremity. *NeuroRehabilitation*. 2011;29(4):373-379.

26. Rinker B, Zoldos J, Weber R V., et al. Use of Processed Nerve Allografts to Repair Nerve Injuries Greater Than 25 mm in the Hand. *Ann Plast Surg*. 2017;78(6S Suppl 5):S292-S295. doi:http://dx.doi.org/10.1097/SAP.0000000000001037

27. Ahmad I, Mir MA, Khan AH. An Evaluation of Different Bridging Techniques for Short Nerve Gaps. *Ann Plast Surg*. 2017;79(5):482-485. doi:http://dx.doi.org/10.1097/SAP.0000000000001207

28. Tadjalli HE, McIntyre FH, Dolynchuk KN, Murray KA. Digital nerve repair: relationship between severity of injury and sensibility recovery. *Ann Plast Surg*. 1995;35(1):36-40. http://ovidsp.ovid.com/ovidweb.cgi?T=JS&PAGE=reference&D=med3&NEWS=N&AN=7574284

29. Novak CB, Mackinnon SE. Correlation of two-point discrimination and hand function following median nerve injury. *Ann Plast Surg*. 1993;31(6):495-498. http://ovidsp.ovid.com/ovidweb.cgi?T=JS&PAGE=reference&D=emed5&NEWS=N&AN=24000558

30. Tadjalli HE, McIntyre FH, Dolynchuk KN, Murray KA. Importance of crossover innervation in digital nerve repair demonstrated by nerve isolation technique. *Ann Plast Surg*. 1995;35(1):32-35. http://ovidsp.ovid.com/ovidweb.cgi?T=JS&PAGE=reference&D=med3&NEWS=N&AN=7574283

31. Meek MF, Coert JH, Wong KH. Recovery of touch after median nerve lesion and subsequent repair. *Microsurgery*. 2003;23(1):2-5. http://ovidsp.ovid.com/ovidweb.cgi?T=JS&PAGE=reference&D=med4&NEWS=N&AN=12616510

32. Walton RL, Brown RE, Matory WEJ, Borah GL, Dolph JL. Autogenous vein graft repair of digital nerve defects in the finger: a retrospective clinical study. *Plast Reconstr Surg*. 1989;84(6):942-944. http://ovidsp.ovid.com/ovidweb.cgi?T=JS&PAGE=reference&D=med3&NEWS=N&AN=2587658

33. Mackinnon SE, Dellon AL. *Surgery of the Peripheral Nerve*. Vol 396. 1st Editio. Thieme Medical Publishers; 1988.

34. Roseén B, Lundborg G. A model instrument for the documentation of outcome after nerve repair. *J Hand Surg Am*. 2000;25(3):535-543. doi:10.1053/jhsu.2000.6458

35. Moberg E. Evaluation of Sensibility in the Hand. Published online 1960. doi:10.1016/S0039-6109(16)36040-6

36. Weinstein S. Fifty years of somatosensory research: From the Semmes-Weinstein Monofilaments to the Weinstein Enhanced Sensory Test. *J Hand Ther*. 1993;6(1):11-22. doi:10.1016/S0894-1130(12)80176-1

37. Taylor KS, Anastakis DJ, Davis KD. Cutting your nerve changes your brain. *Brain*. 2009;132(11):3122-3133. doi:https://dx.doi.org/10.1093/brain/awp231

38. Bertelli JA, Cavalli E, Mendes Lehn VL, Ghizoni MF. Sensory deficits after a radial nerve injury. *Microsurgery*. 2018;38(2):151-156. doi:https://dx.doi.org/10.1002/micr.30161

39. Chen C, Tang P, Zhang X, et al. Treatment of soft-tissue loss with nerve defect in the finger using the boomerang nerve flap. *Plast Reconstr Surg*. 2013;131(1):44e-54e. doi:https://dx.doi.org/10.1097/PRS.0b013e3182729f5e

40. Attal N, Rouaud J, Brasseur L, et al. Systemic lidocaine in pain due to peripheral nerve injury and predictors of response. *Neurology*. 2004;62(2):218-225. http://ovidsp.ovid.com/ovidweb.cgi?T=JS&PAGE=reference&D=emed8&NEWS=N&AN=38167128

41. Vollert J, Attal N, Baron R, et al. Quantitative sensory testing using DFNS protocol in Europe: an evaluation of heterogeneity across multiple centers in patients with peripheral neuropathic pain and healthy subjects. *Pain*. 2016;157(3):750-758. doi:https://dx.doi.org/10.1097/j.pain.0000000000000433

42. Gierthmuhlen J, Maier C, Baron R, et al. Sensory signs in complex regional pain syndrome and peripheral nerve injury. Baron R Binder A, Koroschetz J, Maier C, Richter H, Krumova EK, Westermann A, Tolle T, Berthele A, Sprenger T, Valet M, Munchen TU, Treede RD, Magerl W, Klein T, Birklein F, Geber C, Rolke R, Maihofner C, Azad SC, Beyer A, Huge V, Lauchart M, Birbaumer N GJ, ed. *Pain*. 2012;153(4):765-774. doi:https://dx.doi.org/10.1016/j.pain.2011.11.009

43. Bloom S, Till S, Sonksen P, Smith S. Use of a biothesiometer to measure individual vibration thresholds and their variation in 519 non-diabetic subjects. *Br Med J*. 1984;288(6433):1793-1795. doi:10.1136/bmj.288.6433.1793

44. Lundborg G, Dahlin LB, Lundström R, Necking LE, Strömberg T. Vibrotactile function of the hand in compression and vibration-induced neuropathy: Sensibility index-a new measure. *Scand J Plast Reconstr Surg Hand Surg*. 1992;26(3):275-279. doi:10.3109/02844319209015271

45. Gottrup H, Bach FW, Juhl G, et al. Differential effect of ketamine and lidocaine on spontaneous and mechanical evoked pain in patients with nerve injury pain. *Anesthesiology*. 2006;104(3):527-536. doi:http://dx.doi.org/10.1097/00000542-200603000-00021

46. Witting N, Kupers RC, Svensson P, Jensen TS. A PET activation study of brush-evoked allodynia in patients with nerve injury pain. *Pain*. 2006;120(1-2):145-154. doi:http://dx.doi.org/10.1016/j.pain.2005.10.034

47. Gordh TE, Stubhaug A, Jensen TS, et al. Gabapentin in traumatic nerve injury pain: a randomized, double-blind, placebo-controlled, cross-over, multi-center study. *Pain*. 2008;138(2):255-266. doi:https://dx.doi.org/10.1016/j.pain.2007.12.011

48. Yarnitsky D. Quantitative sensory testing. *Muscle Nerve*. 1997;(February):198-204.

49. Rosén B, Lundborg G. A new tactile gnosis instrument in sensibility testing. *J Hand Ther*. 1998;11(4):251-257. doi:10.1016/S0894-1130(98)80020-3

50. Dellon AL, Kallman CH. Evaluation of functional sensation in the hand. *J Hand Surg Am*. 1983;8(6):865-870. doi:10.1016/S0363-5023(83)80083-5

51. Van Boven RW, Johnson KO. A psychophysical study of the mechanisms of sensory recovery following nerve injury in humans. *Brain*. 1994;117(1):149-167. doi:10.1093/brain/117.1.149

52. Walton R, Finseth F. Nerve grafting in the repair of complicated peripheral nerve trauma. *J Trauma*. 1977;17(10):793-796. http://ovidsp.ovid.com/ovidweb.cgi?T=JS&PAGE=reference&D=med1&NEWS=N&AN=909120

53. Highet W, Holmes W. TRACTION INJURIES TO THE LATERAL POPLITEAL NERVE AND TRACTION INJURIES TO PERIPHERAL NERVES AFTER SUTURE. *Br J S*. 1943;30(119):212-233.

54. Amillo S, Barrios RH, Martinez-Peric R, Losada JI. Surgical treatment of the radial nerve lesions associated with fractures of the humerus. *J Orthop Trauma*. 1993;7(3):211-215. http://ovidsp.ovid.com/ovidweb.cgi?T=JS&PAGE=reference&D=med3&NEWS=N&AN=8326423

55. Meek MF, Coert JH, Robinson PH. Poor results after nerve grafting in the upper extremity: Quo vadis?. *Microsurgery*. 2005;25(5):396-402. doi:10.1002/micr.20137

56. Kalomiri DE, Soucacos PN. Nerve grafting in peripheral nerve microsurgery of the upper extremity. *Microsurgery*. 1994;15(7):506-511. http://ovidsp.ovid.com/ovidweb.cgi?T=JS&PAGE=reference&D=emed5&NEWS=N&AN=24279127

57. Roganovic Z, Pavlicevic G. Difference in recovery potential of peripheral nerves after graft repairs. *Neurosurgery*. 2006;59(3):621-632. doi:http://dx.doi.org/10.1227/01.NEU.0000228869.48866.BD

58. Bai L, Wang T-B, Wang X, et al. Use of nerve elongator to repair short-distance peripheral nerve defects: a prospective randomized study. *Neural Regen Res*. 2015;10(1):79‐83. doi:10.4103/1673-5374.150710

59. Brushart TM. *Nerve Repair*. Oxford University Press; 2011. https://books.google.co.uk/books?id=RNQIfsA2iUUC

60. Vaughn CJ, Raghavan SS, Hansen SL, et al. Obstacles to the Care of Patients With Multicomponent Volar Wrist Lacerations at a County Hospital. *Ann Plast Surg*. 2016;76 Suppl 3(Supplement 3):S238-S240. doi:http://dx.doi.org/10.1097/SAP.0000000000000801

61. Wang E, Inaba K, Byerly S, et al. Optimal timing for repair of peripheral nerve injuries. *J Trauma Acute Care Surg*. 2017;83(5):875-881. doi:http://dx.doi.org/10.1097/TA.0000000000001570

62. Daoutis NK, Gerostathopoulos NE, Efstathopoulos DG, Misitizis DP, Bouchlis GN. Microsurgical reconstruction of large nerve defects using autologous nerve grafts. *Microsurgery*. 1994;15(7):502-505. http://ovidsp.ovid.com/ovidweb.cgi?T=JS&PAGE=reference&D=emed5&NEWS=N&AN=24279126

63. Lin C-H, Mardini S, Levin SL, Lin Y-T, Yeh J-T. Endoscopically assisted sural nerve harvest for upper extremity posttraumatic nerve defects: an evaluation of functional outcomes. *Plast Reconstr Surg*. 2007;119(2):616-626. doi:http://dx.doi.org/10.1097/01.prs.0000253220.60630.99

64. Sakellarides H. A follow-up study of 172 peripheral nerve injuries in the upper extremity in civilians. *J Bone Joint Surg Am*. 1962;44-A:140-148. http://ovidsp.ovid.com/ovidweb.cgi?T=JS&PAGE=reference&D=med1&NEWS=N&AN=14038909

65. Taha A, Taha J. Results of suture of the radial, median, and ulnar nerves after missile injury below the axilla. *J Trauma*. 1998;45(2):335-339. http://ovidsp.ovid.com/ovidweb.cgi?T=JS&PAGE=reference&D=med4&NEWS=N&AN=9715192

66. Samardzic MM, Rasulic LG. Gunshot injuries to the brachial plexus. *J Trauma - Inj Infect Crit Care*. 1997;43(4):645-649. doi:http://dx.doi.org/10.1097/00005373-199710000-00014
