# Supplementary material for: A Quantitative Systematic Review of Clinical Outcome Measure Use in Peripheral Nerve Injury of the Upper Limb
Source: Neurosurgery. 2021 Mar 8;89(1):22–30. doi: 10.1093/neuros/nyab060 (PMC8203424; doi:10.1093/neuros/nyab060)
Supplement: nyab060_Supplemental_Files [file nyab060_supplemental_files.zip › SR Outcome Measures PNI.Supplementary Table 2.docx]

Supplementary Table 2: Motor Outcome Reporting

| Outcome Measure Domains | Outcome Measures | No. of studies reporting outcome measurement | Instrument | Metric | Specific Time points |
| --- | --- | --- | --- | --- | --- |
|  |  |  |  |  |  |
| Motor objective | Dynamometry  (Grip/Pinch Strength) | 9 | 9 | 9 | 1 |
| Motor  Subjective | Manual Muscle Testing (BMRC Scale) | 30 | 30 | 30 | 4 |
|  | Manual Muscle Testing (Louisiana State University (LSU) Scale) | 2 | 2 | 2 | 0 |
|  | Clinical range of motion | 3 | 3 | 3 | 0 |
|  | Functional assessment | 3 | 3 | 3 | 1 |

*Motor Objective*

In contrast to sensory outcome measures, grip and/or pinch strength measured using dynamometry was the only commonly utilised objective measure ^1–9^. It was uniformly well described whilst the specific reporting metric of force in Newtons or kilograms of force (kgf) was used throughout. Williams et al. ^2^ and Taylor et al. ^9^ reported percentage of normal grip strength as compared with the contralateral uninjured side in mixed upper limb nerve injured patients, whereas Cheing et al. ^8^ reported grip, key pinch and oppositional pinch strengths compared to similar measurements in an uninjured control group. Only Taylor et al. ^9^ described specific assessment time points for assessment at 3, 6 and 12 months after surgery, the remaining studies described ranges. In mixed nerve injuries, time points between 1 year and 18 years were used. In three brachial plexus studies a range of 12 – 42 months was used.

*Motor Subjective*

Subjective measures of motor function include Manual Muscle Testing using the British Medical Research Council (MRC) scale for individual muscles which was used in 30 studies ^1–4,6,7,10–33^; the original Highet’s staging of motor recovery ^34^ from which the MRC scale was based, was utilised in four studies ^4,11,16,19^. It was uniformly well described with the same specific measurement used throughout. Time points for assessment were rarely specified, where stated in brachial plexus injuries, the MRC scale was used anywhere between 20-73 months post-operatively. In mixed upper limb nerve injury studies, it was used anywhere between 3 months – 18 years after injury. The Louisiana State University (LSU) scale developed by Kline and Hudson ^35^, modified the MRC scale, helping to differentiate patients in grade 4 and create a specification for each nerve and was used in 2 studies ^36,37^. It was well described, however neither study clarified time points for its use.

Clinical assessment of range of motion was assessed in three studies. Chu et al. ^38^, Hu et al. ^7^ and Baltzer et al. ^24^ assessed range of movement at the shoulder measured in degrees. These were respectively assessed post-surgery at 1-7years, when recovered and between 12-40 months.

Three studies made functional assessments using a variety of scores. Bai et al. ^20^ used the Mayo Elbow Score to assess elbow function at 3, 6 and 12 months after high median, ulnar and radial nerve injuries. Zhou et al. ^39^ used the Upper Extremity Functional Evaluation of the Chinese Hand Surgery Academy to assess functional recovery in mixed upper limb nerve injury patients which was performed 4 weeks post-surgery. Baltzer et al. ^40^ assessed ulnar intrinsic function compared to an uninjured control group at 1 year post-op in patients undergoing end-to-side anterior interosseous nerve to ulnar motor nerve transfer.

References

1. Schreuders TAR, Roebroeck ME, Jaquet J-B, Hovius SER. Long-term outcome of muscle strength in ulnar and median nerve injury: comparing manual muscle strength testing, grip and pinch strength dynamometers and a new intrinsic muscle strength dynamometer. *J Rehabil Med*. 2004;36(6):273-278. doi:http://dx.doi.org/10.1080/16501970410033677

2. Williams HB. A clinical pilot study to assess functional return following continuous muscle stimulation after nerve injury and repair in the upper extremity using a completely implantable electrical system. *Microsurgery*. 1996;17(11):597-605. doi:http://dx.doi.org/10.1002/%28SICI%291098-2752%281996%2917:11%3C597::AID-MICR6%3E3.0.CO;2-M

3. Sungpet A, Suphachatwong C, Kawinwonggowit V. One-fascicle median nerve transfer to biceps muscle in C5 and C6 root avulsions of brachial plexus injury. *Microsurgery*. 2003;23(1):10-13. http://ovidsp.ovid.com/ovidweb.cgi?T=JS&PAGE=reference&D=med4&NEWS=N&AN=12616512

4. Nunley JA, Saies AD, Sandow MJ. Results of interfascicular nerve grafting for radial nerve lesions. *Microsurgery*. 1997;17(8):431-437. doi:http://dx.doi.org/10.1002/%28SICI%291098-2752%281996%2917:8%3C431::AID-MICR3%3E3.0.CO;2-H

5. Martins RS, Siqueira MG, Heise CO, Foroni L, Teixeira MJ. A prospective study comparing single and double fascicular transfer to restore elbow flexion after brachial plexus injury. *Neurosurgery*. 2013;72(5):709-715. doi:https://dx.doi.org/10.1227/NEU.0b013e318285c3f6

6. Bosnjak RF, Dolenc VV, Sepe A, Demsar F, Tindall SC. Force, fatigue, and the cross-sectional area of wrist extensor muscles after radial nerve grafting. *Neurosurgery*. 1992;31(6):1035-1042. http://ovidsp.ovid.com/ovidweb.cgi?T=JS&PAGE=reference&D=emed4&NEWS=N&AN=22370333

7. Hu C-H, Chang TN-J, Lu JC-Y, et al. Comparison of Surgical Strategies between Proximal Nerve Graft and/or Nerve Transfer and Distal Nerve Transfer Based on Functional Restoration of Elbow Flexion: A Retrospective Review of 147 Patients. *Plast Reconstr Surg*. 2018;141(1):68e-79e. doi:http://dx.doi.org/10.1097/PRS.0000000000003935

8. Cheing GLY, Luk MLM. Transcutaneous electrical nerve stimulation for neuropathic pain. *J Hand Surg Am*. 2005;30(1):50-55. doi:10.1016/j.jhsb.2004.08.007

9. Taylor KS, Anastakis DJ, Davis KD, et al. Chronic pain and sensorimotor deficits following peripheral nerve injury. *Pain*. 2010;151(3):582-591. doi:http://dx.doi.org/10.1016/j.pain.2010.06.032

10. Amillo S, Barrios RH, Martinez-Peric R, Losada JI. Surgical treatment of the radial nerve lesions associated with fractures of the humerus. *J Orthop Trauma*. 1993;7(3):211-215. http://ovidsp.ovid.com/ovidweb.cgi?T=JS&PAGE=reference&D=med3&NEWS=N&AN=8326423

11. Samardzic MM, Rasulic LG. Gunshot injuries to the brachial plexus. *J Trauma - Inj Infect Crit Care*. 1997;43(4):645-649. doi:http://dx.doi.org/10.1097/00005373-199710000-00014

12. Jaquet JB, Luijsterburg AJ, Kalmijn S, et al. Median, ulnar, and combined median-ulnar nerve injuries: Functional outcome and return to productivity. *J Trauma - Inj Infect Crit Care*. 2001;51(4):687-692. Accessed August 16, 2018. http://ovidsp.ovid.com/ovidweb.cgi?T=JS&PAGE=reference&D=emed7&NEWS=N&AN=33029285

13. Wang E, Inaba K, Byerly S, et al. Optimal timing for repair of peripheral nerve injuries. *J Trauma Acute Care Surg*. 2017;83(5):875-881. doi:http://dx.doi.org/10.1097/TA.0000000000001570

14. Meek MF, Coert JH, Robinson PH. Poor results after nerve grafting in the upper extremity: Quo vadis?. *Microsurgery*. 2005;25(5):396-402. doi:10.1002/micr.20137

15. Battiston B, Tos P, Cushway TR, Geuna S. Nerve repair by means of vein filled with muscle grafts I. Clinical results. *Microsurgery*. 2000;20(1):32-36. doi:http://dx.doi.org/10.1002/%28SICI%291098-2752%282000%2920:1%3C32::AID-MICR6%3E3.0.CO;2-D

16. Mailander P, Berger A, Schaller E, et al. Results of primary nerve repair in the upper extremity. *Microsurgery*. 1989;10(2):147-150. http://ovidsp.ovid.com/ovidweb.cgi?T=JS&PAGE=reference&D=emed4&NEWS=N&AN=19181477

17. Kalomiri DE, Soucacos PN. Nerve grafting in peripheral nerve microsurgery of the upper extremity. *Microsurgery*. 1994;15(7):506-511. http://ovidsp.ovid.com/ovidweb.cgi?T=JS&PAGE=reference&D=emed5&NEWS=N&AN=24279127

18. Daoutis NK, Gerostathopoulos NE, Efstathopoulos DG, Misitizis DP, Bouchlis GN. Microsurgical reconstruction of large nerve defects using autologous nerve grafts. *Microsurgery*. 1994;15(7):502-505. http://ovidsp.ovid.com/ovidweb.cgi?T=JS&PAGE=reference&D=emed5&NEWS=N&AN=24279126

19. Becker M, Lassner F, Fansa H, Mawrin C, Pallua N. Refinements in nerve to muscle neurotization. *Muscle Nerve*. 2002;26(3):362-366. http://ovidsp.ovid.com/ovidweb.cgi?T=JS&PAGE=reference&D=med4&NEWS=N&AN=12210365

20. Bai L, Wang T-B, Wang X, et al. Use of nerve elongator to repair short-distance peripheral nerve defects: a prospective randomized study. *Neural Regen Res*. 2015;10(1):79‐83. doi:10.4103/1673-5374.150710

21. Roganovic Z, Pavlicevic G. Difference in recovery potential of peripheral nerves after graft repairs. *Neurosurgery*. 2006;59(3):621-632. doi:http://dx.doi.org/10.1227/01.NEU.0000228869.48866.BD

22. Bertelli JA, Ghizoni MF. Brachial plexus avulsion injury repairs with nerve transfers and nerve grafts directly implanted into the spinal cord yield partial recovery of shoulder and elbow movements. *Neurosurgery*. 2003;52(6):1385-1390. http://ovidsp.ovid.com/ovidweb.cgi?T=JS&PAGE=reference&D=med4&NEWS=N&AN=12762883

23. Lin C-H, Mardini S, Levin SL, Lin Y-T, Yeh J-T. Endoscopically assisted sural nerve harvest for upper extremity posttraumatic nerve defects: an evaluation of functional outcomes. *Plast Reconstr Surg*. 2007;119(2):616-626. doi:http://dx.doi.org/10.1097/01.prs.0000253220.60630.99

24. Ko JH, Baltzer HL, Kircher MF, et al. Discussion: A Comparison of Outcomes of Triceps Motor Branch-to-Axillary Nerve Transfer or Sural Nerve Interpositional Grafting for Isolated Axillary Nerve Injury. *Plast Reconstr Surg*. 2016;138(2):265e-7e. doi:https://dx.doi.org/10.1097/PRS.0000000000002368

25. Boonstra A, Van Weerden T, Eisma W, Pahlplatz V, Oosterhuis H. The effect of low-frequency electrical stimulation on denervation atrophy in man. *Scand J Rehabil Med*. 1987;19(3):127-134.

26. Sakellarides H. A follow-up study of 172 peripheral nerve injuries in the upper extremity in civilians. *J Bone Joint Surg Am*. 1962;44-A:140-148. http://ovidsp.ovid.com/ovidweb.cgi?T=JS&PAGE=reference&D=med1&NEWS=N&AN=14038909

27. Young C, Hudson A, Richards R. Operative treatment of palsy of the posterior interosseous nerve of the forearm. *J Bone Joint Surg Am*. 1990;72(8):1215-1219. http://ovidsp.ovid.com/ovidweb.cgi?T=JS&PAGE=reference&D=med3&NEWS=N&AN=2204631

28. Walton R, Finseth F. Nerve grafting in the repair of complicated peripheral nerve trauma. *J Trauma*. 1977;17(10):793-796. http://ovidsp.ovid.com/ovidweb.cgi?T=JS&PAGE=reference&D=med1&NEWS=N&AN=909120

29. Daneyemez M, Solmaz I, Izci Y, et al. Prognostic factors for the surgical management of peripheral nerve lesions. *Tohoku J Exp Med*. 2005;205(3):269-275. doi:http://dx.doi.org/10.1620/tjem.205.269

30. Frueh FS, Ho M, Schiller A, et al. Magnetic Resonance Neurographic and Clinical Long-Term Results After Oberlin’s Transfer for Adult Brachial Plexus Injuries. *Ann Plast Surg*. 2017;78(1):67-72. http://ovidsp.ovid.com/ovidweb.cgi?T=JS&PAGE=reference&D=emed18&NEWS=N&AN=618707587

31. Ahmad I, Mir MA, Khan AH. An Evaluation of Different Bridging Techniques for Short Nerve Gaps. *Ann Plast Surg*. 2017;79(5):482-485. doi:http://dx.doi.org/10.1097/SAP.0000000000001207

32. Vaughn CJ, Raghavan SS, Hansen SL, et al. Obstacles to the Care of Patients With Multicomponent Volar Wrist Lacerations at a County Hospital. *Ann Plast Surg*. 2016;76 Suppl 3(Supplement 3):S238-S240. doi:http://dx.doi.org/10.1097/SAP.0000000000000801

33. Lundborg G, Rosén B, Dahlin L, Danielsen N, Holmberg J. Tubular versus conventional repair of median and ulnar nerves in the human forearm: early results from a prospective, randomized, clinical study. *J Hand Surg Am*. 1997;22(1 CC-Child Health CC-Bone, Joint and Muscle Trauma CC-Neuromuscular):99‐106. doi:10.1016/S0363-5023(05)80188-1

34. Highet W, Holmes W. TRACTION INJURIES TO THE LATERAL POPLITEAL NERVE AND TRACTION INJURIES TO PERIPHERAL NERVES AFTER SUTURE. *Br J S*. 1943;30(119):212-233.

35. Kim D, Midha R, Murovic JA, Spinner R, Teil R. *Kline and Hudson’s Nerve Injuries*. 2nc Editio. Saunders Elsevier; 2007. doi:10.2106/00004623-199601000-00029

36. Ferreira SR, Martins RS, Siqueira MG. Correlation between motor function recovery and daily living activity outcomes after brachial plexus surgery. *Arq Neuropsiquiatr*. 2017;75(9):631-634. doi:https://dx.doi.org/10.1590/0004-282X20170090

37. Sulaiman OAR, Kim DD, Burkett C, Kline DG. Nerve transfer surgery for adult brachial plexus injury: a 10-year experience at Louisiana State University. *Neurosurgery*. 2009;65(4 Suppl):A55-62. doi:https://dx.doi.org/10.1227/01.NEU.0000341165.83218.AC

38. Chu B, Wang H, Chen L, et al. Dual Nerve Transfers for Restoration of Shoulder Function After Brachial Plexus Avulsion Injury. *Ann Plast Surg*. 2016;76(6):668-673. doi:https://dx.doi.org/10.1097/SAP.0000000000000747

39. Zhou J-M, Gu Y-D, Xu X-J, Zhang S-Y, Zhao X. Clinical research of comprehensive rehabilitation in treating brachial plexus injury patients. *Chin Med J (Engl)*. 2012;125(14):2516-2520. http://ovidsp.ovid.com/ovidweb.cgi?T=JS&PAGE=reference&D=med7&NEWS=N&AN=22882932

40. Baltzer H, Woo A, Oh C, Moran SL. Comparison of Ulnar Intrinsic Function following Supercharge End-to-Side Anterior Interosseous-to-Ulnar Motor Nerve Transfer: A Matched Cohort Study of Proximal Ulnar Nerve Injury Patients. *Plast Reconstr Surg*. 2016;138(6):1264-1272. http://ovidsp.ovid.com/ovidweb.cgi?T=JS&PAGE=reference&D=med8&NEWS=N&AN=27879594
