# Supplementary material for: A Quantitative Systematic Review of Clinical Outcome Measure Use in Peripheral Nerve Injury of the Upper Limb
Source: Neurosurgery. 2021 Mar 8;89(1):22–30. doi: 10.1093/neuros/nyab060 (PMC8203424; doi:10.1093/neuros/nyab060)
Supplement: nyab060_Supplemental_Files [file nyab060_supplemental_files.zip › SR Outcome Measures PNI.Supplementary Table 3.docx]

Supplementary Table 3: Sensorimotor Function Outcome Reporting

| Outcome Measures | No. of studies reporting outcome measurement | Instrument | Metric | Specific Time points |
| --- | --- | --- | --- | --- |
|  |  |  |  |  |
| Moberg’s Pick-up Test | 1 | 1 | 1 | 0 |
| Minnesota Rate of Manipulation Test | 1 | 1 | 1 | 0 |
| Southampton Hand Assessment Procedure (SHAP) | 1 | 1 | 1 | 0 |
| Grooved Pegboard | 1 | 1 | 0 | 0 |

Four separate sensorimotor tests were employed to assess sensorimotor function. Tadjalli et al. used Moberg’s Pick-up Test ^1^, whilst Nunley et al. ^2^ used the Minnesota Rate of Manipulation Test and Taylor et al. ^3^ used the Southampton Hand Assessment Procedure (SHAP) and the grooved pegboard test. The Minnesota Rate of Manipulation Test, Moberg’s Pick-up Test and the grooved pegboard test were timed tests and thus reported in seconds required to complete the relevant tasks. Contralateral controls were used as the comparator whilst all three studies performed the measures at a considerable time after surgery/injury, Tadjalli et al ^1^ at a mean of 35 months (16 - 87 months), Nunley et al. ^2^ at a mean of 38 months (12 months – 10 years) and Taylor et al. ^3^ at 5 years (+/- 3 years). Taylor et al. ^3^ performed the SHAP test at 5 (+/- 3) years after surgery with a score out of 100 recorded.

References

1. Tadjalli HE, McIntyre FH, Dolynchuk KN, Murray KA. Digital nerve repair: relationship between severity of injury and sensibility recovery. *Ann Plast Surg*. 1995;35(1):36-40. http://ovidsp.ovid.com/ovidweb.cgi?T=JS&PAGE=reference&D=med3&NEWS=N&AN=7574284.

2. Nunley JA, Saies AD, Sandow MJ. Results of interfascicular nerve grafting for radial nerve lesions. *Microsurgery*. 1997;17(8):431-437. doi:http://dx.doi.org/10.1002/%28SICI%291098-2752%281996%2917:8%3C431::AID-MICR3%3E3.0.CO;2-H

3. Taylor KS, Anastakis DJ, Davis KD. Chronic pain and sensorimotor deficits following peripheral nerve injury. *Pain*. 2010;151(3):582-591. doi:http://dx.doi.org/10.1016/j.pain.2010.06.032
