# Supplementary material for: A Quantitative Systematic Review of Clinical Outcome Measure Use in Peripheral Nerve Injury of the Upper Limb
Source: Neurosurgery. 2021 Mar 8;89(1):22–30. doi: 10.1093/neuros/nyab060 (PMC8203424; doi:10.1093/neuros/nyab060)
Supplement: nyab060_Supplemental_Files [file nyab060_supplemental_files.zip › SR Outcome Measures PNI.Supplementary Table 4.docx]

Supplementary Table 4: Psychology and Wellbeing Outcome Reporting

| Outcome Measures | No. of studies reporting outcome measurement | Instrument | Metric | Specific Time points |
| --- | --- | --- | --- | --- |
|  |  |  |  |  |
| Hospital Anxiety and Depression Scale (HADS) | 2 | 2 | 2 | 0 |
| Post-traumatic Stress Disorder (PTSD) Checklist – civilian version (PCL-C) | 1 | 1 | 1 | 0 |
| Neuroticism, Extraversion and Openness - Five Factor Inventory (NEO-FFI) | 2 | 2 | 2 | 1 |
| Beck Depression Inventory | 1 | 1 | 1 | 0 |
| Sense of Coherence 13-item scale | 1 | 1 | 1 | 0 |

Novak et al. ^1^ and Nashold et al. ^2^ used a patient-reported outcome measure (PROM) called the Hospital Anxiety and Depression Scale (HADS) to assess psychological distress related to brachial plexus (between 6 months – 15 years post-operatively) or significant mixed upper limb nerve injury (at a mean of 18 months +/- 18 months post-operatively) respectively. Novak et al. ^1^ also used another PROM, the Post-traumatic Stress Disorder (PTSD) Checklist – civilian version (PCL-C), a self-report rating scale for PTSD in the same cohort of patients at similar time points. Goswami et al. ^3^ and Taylor et al. ^4^ used the Neuroticism, Extraversion and Openness - Five Factor Inventory (NEO-FFI) (a psychological personality inventory providing measures of 5 basic personality factors) to assess the effect of personality on pain and cold pain threshold relationships. Goswami et al assessed patients at 2-3 weeks after surgery and after one-year post-injury, whilst Taylor et al. assessed patients at a mean of 5 years after injury. Ciaramitaro et al. ^5^ utilised the Beck Depression Inventory to assess psychological quality of life at 99 days (range 25 – 150 days) after surgery.

Only one study assessed mental wellbeing, Chemnitz et al. ^6^ used the Sense of Coherence 13-item scale ^7^ which assesses a patients’ outlook on life in terms of their ability to see the world as comprehensible, manageable and meaningful. Chemnitz et al used this questionnaire in a long-term (30+ years) follow-up study of patients with forearm/arm (mixed) nerve injuries.

References

1. Novak CB, Anastakis DJ, Beaton DE, et al. Biomedical and psychosocial factors associated with disability after peripheral nerve injury. *J Bone Jt Surg - Ser A*. 2011;93(10):929-936. doi:http://dx.doi.org/10.2106/JBJS.J.00110

2. Nashold BSJ, Goldner JL, Mullen JB, Bright DS. Long-term pain control by direct peripheral-nerve stimulation. *J Bone Joint Surg Am*. 1982;64(1):1-10. http://ovidsp.ovid.com/ovidweb.cgi?T=JS&PAGE=reference&D=med2&NEWS=N&AN=6976348.

3. Goswami R, Anastakis DJ, Katz J, Davis KD. A longitudinal study of pain, personality, and brain plasticity following peripheral nerve injury. *Pain*. 2016;157(3):729-739. doi:http://dx.doi.org/10.1097/j.pain.0000000000000430

4. Taylor KS, Anastakis DJ, Davis KD. Chronic pain and sensorimotor deficits following peripheral nerve injury. *Pain*. 2010;151(3):582-591. doi:http://dx.doi.org/10.1016/j.pain.2010.06.032

5. Ciaramitaro P, Mondelli M, Logullo F, et al. Traumatic peripheral nerve injuries: Epidemiological findings, neuropathic pain and quality of life in 158 patients. *J Peripher Nerv Syst*. 2010;15(2):120-127. doi:http://dx.doi.org/10.1111/j.1529-8027.2010.00260.x

6. Chemnitz A, Dahlin LB. Consequences and adaptation in daily life - Patients’ experiences three decades after a nerve injury sustained in adolescence. *BMC Musculoskelet Disord*. 2013;14:252. doi:http://dx.doi.org/10.1186/1471-2474-14-252

7. Eriksson M, Lindström B. Validity of Antonovsky’s sense of coherence scale: A systematic review. *J Epidemiol Community Health*. 2005;59(6):460-466. doi:10.1136/jech.2003.018085
