# Supplementary material for: A Quantitative Systematic Review of Clinical Outcome Measure Use in Peripheral Nerve Injury of the Upper Limb
Source: Neurosurgery. 2021 Mar 8;89(1):22–30. doi: 10.1093/neuros/nyab060 (PMC8203424; doi:10.1093/neuros/nyab060)
Supplement: nyab060_Supplemental_Files [file nyab060_supplemental_files.zip › SR Outcome Measures PNI.Supplementary Table 5.docx]

Supplementary Table 5: Disability Outcome Reporting

| Outcome Measures | No. of studies reporting outcome measurement | Instrument | Metric | Specific Time points |
| --- | --- | --- | --- | --- |
|  |  |  |  |  |
| Disabilities of the Arm, Shoulder and Hand (DASH) PROM | 8 | 8 | 8 | 3 |
| Occupational Performance PROM | 1 | 1 | 1 | 0 |
| Groningen Activity Restriction Scale PROM | 1 | 1 | 1 | 0 |
| The modified Rankin Scale PROM | 1 | 1 | 1 | 0 |
| Overall Neuropathy Limitations Scale (OLNS) PROM | 1 | 1 | 1 | 0 |

The Disabilities of the Arm, Shoulder and Hand (DASH) PROM ^1^ was the most commonly used disability outcome measure with a total of 8 studies using this tool ^2–9^. Where specified it was used monthly (1-6 months) after hand sensory nerve repair by Wong et al. ^2^; at 3, 6 and 12-months by Bai et al. ^8^ in mixed upper limb nerve injuries and at 1 year after nerve transfer surgery for brachial plexus injured patients by Ferreira et al. ^5^. Ducic et al. ^3^ used the abbreviated version, the quick DASH score after 2 years in patients with mixed upper limb nerve injuries.

Tadjalli et al. ^10^ used an occupational performance PROM ^11^ developed by Canadian Occupational Therapists to assess the effect of digital nerve injury and repair on self-care, leisure and productivity. It was used at a mean of 35 months post-operatively (range 16 - 87 months). Meiners et al. ^12^ used the Groningen Activity Restriction Scale PROM to assess the impact of digital and mixed upper limb nerve injuries (directly repaired) upon activities of daily living. It was used at a mean of 2 years post-operatively. Ciaramitaro et al. ^13^ used the modified Rankin Scale PROM and the Overall Neuropathy Limitations Scale (OLNS) PROM to assess degree of disability, in addition to the DASH score. These were both used at a mean of 99 days (range 25 – 150 days) post-operatively in brachial plexus injured patients.

References

1. Institute for Work and Health. Full DASH. 2006:100. doi:papers3://publication/uuid/4B4FE492-834E-4652-8870-395E0071D0B2

2. Wong JN, Olson JL, Morhart MJ, Chan KM. Electrical stimulation enhances sensory recovery: a randomized controlled trial. *Ann Neurol*. 2015;77(6):996‐1006. doi:10.1002/ana.24397

3. Ducic I, Fu R, Iorio ML. Innovative treatment of peripheral nerve injuries: combined reconstructive concepts. *Ann Plast Surg*. 2012;68(2):180-187. doi:https://dx.doi.org/10.1097/SAP.0b013e3182361b23

4. Frueh FS, Ho M, Schiller A, et al. Magnetic Resonance Neurographic and Clinical Long-Term Results After Oberlin’s Transfer for Adult Brachial Plexus Injuries. *Ann Plast Surg*. 2017;78(1):67-72. http://ovidsp.ovid.com/ovidweb.cgi?T=JS&PAGE=reference&D=emed18&NEWS=N&AN=618707587.

5. Ferreira SR, Martins RS, Siqueira MG. Correlation between motor function recovery and daily living activity outcomes after brachial plexus surgery. *Arq Neuropsiquiatr*. 2017;75(9):631-634. doi:https://dx.doi.org/10.1590/0004-282X20170090

6. Chemnitz A, Dahlin LB. Consequences and adaptation in daily life - Patients’ experiences three decades after a nerve injury sustained in adolescence. *BMC Musculoskelet Disord*. 2013;14:252. doi:http://dx.doi.org/10.1186/1471-2474-14-252

7. Novak CB, Anastakis DJ, Beaton DE, et al. Biomedical and psychosocial factors associated with disability after peripheral nerve injury. *J Bone Jt Surg - Ser A*. 2011;93(10):929-936. doi:http://dx.doi.org/10.2106/JBJS.J.00110

8. Bai L, Wang T-B, Wang X, et al. Use of nerve elongator to repair short-distance peripheral nerve defects: a prospective randomized study. *Neural Regen Res*. 2015;10(1):79‐83. doi:10.4103/1673-5374.150710

9. Ko JH, Baltzer HL, Kircher MF, et al. Discussion: A Comparison of Outcomes of Triceps Motor Branch-to-Axillary Nerve Transfer or Sural Nerve Interpositional Grafting for Isolated Axillary Nerve Injury. *Plast Reconstr Surg*. 2016;138(2):265e-7e. doi:https://dx.doi.org/10.1097/PRS.0000000000002368

10. Tadjalli HE, McIntyre FH, Dolynchuk KN, Murray KA. Digital nerve repair: relationship between severity of injury and sensibility recovery. *Ann Plast Surg*. 1995;35(1):36-40. http://ovidsp.ovid.com/ovidweb.cgi?T=JS&PAGE=reference&D=med3&NEWS=N&AN=7574284.

11. Townsend E, Brintnell S, Staisey N. Developing Guidelines for Client-Centred Occupational Therapy Practice. *Can J Occup Ther*. 1990;57(2):69-76. doi:10.1177/000841749005700205

12. Meiners P, Coert J, Robinson P, Meek M. Impairment and employment issues after nerve repair in the hand and forearm. *Disabil Rehabil*. 2005;27(11):617-623.

13. Ciaramitaro P, Mondelli M, Logullo F, et al. Traumatic peripheral nerve injuries: Epidemiological findings, neuropathic pain and quality of life in 158 patients. *J Peripher Nerv Syst*. 2010;15(2):120-127. doi:http://dx.doi.org/10.1111/j.1529-8027.2010.00260.x
