# Supplementary material for: A Quantitative Systematic Review of Clinical Outcome Measure Use in Peripheral Nerve Injury of the Upper Limb
Source: Neurosurgery. 2021 Mar 8;89(1):22–30. doi: 10.1093/neuros/nyab060 (PMC8203424; doi:10.1093/neuros/nyab060)
Supplement: nyab060_Supplemental_Files [file nyab060_supplemental_files.zip › SR Outcome Measures PNI.Supplementary Table 6.docx]

Supplementary Table 6: Quality of Life Outcome Reporting

| Outcome Measures | No. of studies reporting outcome measurement | Instrument | Metric | Specific Time points |
| --- | --- | --- | --- | --- |
|  |  |  |  |  |
| The Short Form – 36 PROM | 4 | 4 | 4 | 2 |
| The Clinical Global Impressions (CGI) scale | 1 | 1 | 1 | 1 |
| The Patient Global Impression of Change (PGIC) scale | 3 | 3 | 3 | 3 |
| Visual analogue scale (VAS) to assess the impact of nerve injury on education and leisure activities | 1 | 1 | 1 | 0 |

The Short Form – 36 PROM was the most commonly utilised assessment of quality of life and was used in 4 studies ^1–4^. Gordh et al. ^1^ used it in mixed nerve injuries, whereas Colini Baldeschi et al. ^2^, Ciaramitaro et al. ^3^ and Santana et al. ^4^ used the form in brachial plexus injured patients. Where specified it was used at 6-months post-operatively.

The Clinical Global Impressions (CGI) scale ^5^ comprises three measures: severity of illness, global improvement (both of which are measured through a seven-point scale) and efficacy index. It is intended to be used by a clinician to assess the patient’s global functioning prior to and after an intervention. Gordh et al. ^1^ used the CGI to assess the effect of gabapentin in traumatic nerve injury pain in patients with mixed upper limb nerve injury. The Patient Global Impression of Change (PGIC) scale, which is the patient-reported outcome counterpart to the Clinical Global Impressions (CGI) scale ^5^, consists of one item taken from the CGI and adapted to the patient to assess if there has been an improvement or decline in clinical status. It was used in three studies ^1,3,6^ two involving mixed upper limb nerve injuries and one involving brachial plexus nerve injuries and where specified it was used at 1, 3 and 6 months after surgery.

Chemitz et al. ^7^ used a visual analogue scale (VAS) to assess the impact of the nerve injury on patients’ education and leisure activities which was used at a median of 31 years (range 23-40 years) after injury/surgery.

References

1. Gordh TE, Stubhaug A, Jensen TS, et al. Gabapentin in traumatic nerve injury pain: a randomized, double-blind, placebo-controlled, cross-over, multi-center study. *Pain*. 2008;138(2):255-266. doi:https://dx.doi.org/10.1016/j.pain.2007.12.011

2. Colini Baldeschi G, Dario A, De Carolis G, et al. Peripheral Nerve Stimulation in the Treatment of Chronic Pain Syndromes From Nerve Injury: A Multicenter Observational Study. *Neuromodulation*. 2017;20(4):369-374. doi:http://dx.doi.org/10.1111/ner.12539

3. Ciaramitaro P, Mondelli M, Logullo F, et al. Traumatic peripheral nerve injuries: Epidemiological findings, neuropathic pain and quality of life in 158 patients. *J Peripher Nerv Syst*. 2010;15(2):120-127. doi:http://dx.doi.org/10.1111/j.1529-8027.2010.00260.x

4. Santana MVB, Bina MT, Paz MG, et al. High prevalence of neuropathic pain in the hand of patients with traumatic brachial plexus injury: A cross-sectional study. *Arq Neuropsiquiatr*. 2016;74(11):895-901. doi:http://dx.doi.org/10.1590/0004-282X20160149

5. Busner J, Targum SD. The clinical global impressions scale: applying a research tool in clinical practice. *Psychiatry (Edgmont)*. 2007;4(7):28-37. http://www.ncbi.nlm.nih.gov/pubmed/20526405. Accessed May 10, 2020.

6. Vollert J, Attal N, Baron R, et al. Quantitative sensory testing using DFNS protocol in Europe: an evaluation of heterogeneity across multiple centers in patients with peripheral neuropathic pain and healthy subjects. *Pain*. 2016;157(3):750-758. doi:https://dx.doi.org/10.1097/j.pain.0000000000000433

7. Chemnitz A, Dahlin LB. Consequences and adaptation in daily life - Patients’ experiences three decades after a nerve injury sustained in adolescence. *BMC Musculoskelet Disord*. 2013;14:252. doi:http://dx.doi.org/10.1186/1471-2474-14-252
