# Supplementary material for: A Quantitative Systematic Review of Clinical Outcome Measure Use in Peripheral Nerve Injury of the Upper Limb
Source: Neurosurgery. 2021 Mar 8;89(1):22–30. doi: 10.1093/neuros/nyab060 (PMC8203424; doi:10.1093/neuros/nyab060)
Supplement: nyab060_Supplemental_Files [file nyab060_supplemental_files.zip › SR Outcome Measures PNI.Supplementary Table 7.docx]

Supplementary Table 7: Pain and Discomfort outcome reporting

| Outcome Measure Sub-Domains | Outcome Measures | No. of studies reporting outcome | Instrument | Metric | Specific Time points |
| --- | --- | --- | --- | --- | --- |
| Pain Intensity Scales | Visual Analogue Scale  (VAS) | 12 | 12 | 12 | 10 |
|  | Numeric pain  rating scale (NRS) | 5 | 5 | 5 | 5 |
| Pain Intensity Questionnaires (Patient-Reported) | Short-Form McGill Pain Questionnaire (SF-MPQ) | 3 | 3 | 3 | 2 |
|  | Mainz Pain Centre Questionnaire | 1 | 1 | 0 | 1 |
| Allodynia | Mechanical/ Dynamic  Allodynia | 9 | 9 | 9 | 7 |
|  | Cold allodynia | 6 | 6 | 6 | 1 |
| Pain  Thresholds | Thermal pain  thresholds (heat and/or cold) | 8 | 8 | 8 | 3 |
|  | Tactile pain  threshold | 7 | 7 | 7 | 2 |
|  | Pressure pain threshold | 6 | 6 | 6 | 1 |
| Evoked Pain Intensity | Pinprick- evoked Pain | 3 | 3 | 3 | 3 |
|  | Wind-up ratio | 3 | 3 | 3 | 1 |
| Psychological Impact of Pain | Pain Catastrophizing Scale | 3 | 3 | 3 | 1 |
| Neuropathic Pain Questionnaires | Clinician  administered DN4 questionnaire | 2 | 2 | 2 | 0 |
|  | Neuropathic Pain Symptom  Inventory (NPSI) (Patient-Reported) | 1 | 1 | 1 | 1 |

The patient-reported Visual Analogue Scale (VAS) was the most common measure used to assess pain after peripheral nerve injury and was used in 12 studies ^123456789101112^. It was well described in all studies. Where specified in brachial plexus nerve injury it was assessed at 1, 3 and 6 months after surgery, Ciaramitaro et al. ^9^ used most frequently at 3 months after surgery, whereas Santana et al. ^10^ used it up to 18 months after injury.

The patient-reported numeric pain rating scale was used to assess pain in 5 studies ^2,5,13–15^. It was well described in all studies. It is 0 - 10 scale used to measure pain intensity. Specific time points for its use were given in all studies. Gordh et al. ^5^ used it pre- and post-operatively in mixed nerve injury patients whereas Nikolajsen ^13^ used it 1 week before neuroma removal surgery and 1, 3 and 6 months after surgery.

Two patient-reported pain intensity questionnaires were used to assess pain intensity. Novak et al. ^16^, Goswami et al. ^17^ and Witting et al. ^18^ used the Short-Form McGill Pain Questionnaire (SF-MPQ) whereas Eisenberg et al. ^2^ used the Mainz Pain Centre Questionnaire. The SF-MPQ was well described in all three studies. Novak et al. used the questionnaire in a long term follow up study of brachial plexus patients between 6 months – 15 years after injury, whereas Goswami et al. used it at 2-3 weeks after surgery and after 1-year post injury in mixed nerve injury patients.

Mechanical (or dynamic) allodynia (painful hypersensitivity) was assessed in 9 studies ^1,7,11,12,15,17–20^. All studies used a brush as the stimulus with 5 of these studies ^1,5,20–22^ measuring the pain response using the VAS and one study ^23^ using the numerical pain rating scale. Time points for assessment were specified in 7 studies – all mixed upper limb nerve injury studies with completely different time points used in each study.

Cold allodynia was assessed in 6 studies ^1,14,16,19,24,25^ Chemnitz et al. ^24^ and Novak et al. ^16^ utilised the patient-reported cold intolerance symptom severity (CISS) questionnaire ^26^, which defines the severity of cold induced symptoms, to assess cold allodynia. Lundborg et al. ^19^, Chen et al. ^25^ and Taylor et al. ^14^ assessed cold intolerance using a patient-reported score of 1-4 (minor/moderate/disturbing/hindrance to function) or scale (0 – 10). Where specified assessments were made at 3, 6 and 12 months after surgery after mixed upper limb nerve injury.

Thermal pain thresholds (heat and/or cold) were assessed in 8 studies ^1,4,13,14,17,18,20,23^. Gottrup et al. ^1^, Nikolajsen et al. ^13^, Attal et al. ^4^ and Witting et al. ^18^ used a thermotester (SOMEDIC,Hörby, Sweden)) device whereas Taylor et al. ^14^, Goswami et al. ^17^ and Vollert et al. ^20^ used a computer-controlled (Peltier- (TSA-II NeuroSensory Analyzer, Medoc Ltd., Israel) to assess thermal pain threshold. Only Attal et al. ^4^ used a patient-reported visual analogue scale to further quantify the degree of pain felt. Two mixed upper limb nerve injury studies ^13,17^ specified time points for follow up at before and 3 months after surgery and 2-3 weeks and after 1 year after injury.

Tactile pain threshold was assessed in 7 studies ^1,12,13,15,18,20,23^. Five studies used von Frey filaments to induce the stimulus, whereas Gierthmuhlen et al. ^23^ and Vollert et al. ^20^ used pin prick stimuli. Both of these studies used a patient-reported visual analogue scale to measure the effect of the stimulus, whilst Kalliomaki et al. ^15^ used a numerical rating scale. Only two studies specified time points for assessment ^12,13^ at before and 3 months after surgery and before and after treatment analysing the effect of systemic adenosine infusion to reduce an area of tactile allodynia in neuropathic pain following peripheral nerve injury.

Pressure pain threshold was utilised in 6 studies ^1,7,13,18,20,23^. Gottrup et al. ^1^, Nikolajsen et al. ^13^, Bouhassira et al. ^7^ and Witting et al. ^18^ used a pressure algometer to quantify the pressure threshold at which pain was felt, whereas Gierthmuhlen et al. ^23^ and Vollert et al. ^20^ used a pressure gauge device. Where specified assessments were made before and at 3 months after surgery. However, 2 studies made assessment more than 5 years and up to 10 years after injury.

Pin-prick evoked pain was used in 3 studies ^1,3,13^. A nylon filament or von Frey hair was used to elicit pain and Gottrup et al. ^3^ used a patient-reported visual analogue score to measure the pain response. Nikolajsen ^13^ used this outcome before and 3 month after neuroma excision, whereas Gottrup et al. ^1^ used it to assess the effect of ketamine and lidocaine on mechanical evoked pain after peripheral nerve injury.

The wind-up ratio was used in 3 studies ^18,20,23^ and involves a series of repetitive pin-prick stimuli which generates increased pain intensity over time. The pain intensity was assessed using a patient-reported visual analogue score in all studies and was assessed prior to positron emission tomography scanning of the brain in patients with nerve injury pain by Witting et al. ^18^, Vollert et al. ^20^ and Gierthmuhlen et al. ^23^ used it after 5 years and up to 10 years respectively in patients with neuropathic pain after nerve injury.

The Pain Catastrophizing scale is a patient-reported outcome measure used to assess the psychological impact of pain ^27^ and was used in 3 studies ^14,16,17^. It was well described in all studies. Only one mixed nerve injury study specified the time points for assessment ^17^ at 2-3 weeks after surgery and after 1 year post injury. Novak et al. ^16^ assessed brachial nerve injured patients between 6 months and 15 years after their injuries, whereas Taylor et al. ^14^ assessed patients at 5 +/- 3 years after injury.

Neuropathic pain was assessed using the clinician administered (DN4) questionnaire by Ciaramitaro et al. ^9^ and Satana et al. ^10^ in brachial plexus injured patients and using the Neuropathic Pain Symptom Inventory (NPSI) by Kalliomaki et al. ^15^ in mixed upper limb nerve injured patients. The clinician administered (DN4) questionnaire ^28^ consists of sensory descriptors and signs related to bedside sensory examination, whereas the NPSI is a patient-reported outcome measure assessing the symptoms of neuropathic pain ^29^. All studies presented the scores of the questionnaires. Where described, time points for assessment were given in ranges. Ciaramitaro et al.^. 9^ assessed patients at 99 days (range 25-150) and Santana et al. ^10^ at 78 +/- 88 weeks post injury.

References

1. Gottrup H, Bach FW, Juhl G, et al. Differential effect of ketamine and lidocaine on spontaneous and mechanical evoked pain in patients with nerve injury pain. *Anesthesiology*. 2006;104(3):527-536. doi:http://dx.doi.org/10.1097/00000542-200603000-00021

2. Eisenberg E, Waisbrod H, Gerbershagen HU. Long-term peripheral nerve stimulation for painful nerve injuries. *Clin J Pain*. 2004;20(3):143-146. doi:http://dx.doi.org/10.1097/00002508-200405000-00003

3. Gottrup H, Kristensen AD, Bach FW, Jensen TS. Aftersensations in experimental and clinical hypersensitivity. *Pain*. 2003;103(1-2):57-64. http://ovidsp.ovid.com/ovidweb.cgi?T=JS&PAGE=reference&D=med4&NEWS=N&AN=12749959

4. Attal N, Rouaud J, Brasseur L, et al. Systemic lidocaine in pain due to peripheral nerve injury and predictors of response. *Neurology*. 2004;62(2):218-225. http://ovidsp.ovid.com/ovidweb.cgi?T=JS&PAGE=reference&D=emed8&NEWS=N&AN=38167128

5. Gordh TE, Stubhaug A, Jensen TS, et al. Gabapentin in traumatic nerve injury pain: a randomized, double-blind, placebo-controlled, cross-over, multi-center study. *Pain*. 2008;138(2):255-266. doi:https://dx.doi.org/10.1016/j.pain.2007.12.011

6. Scadding JW, Wall PD, Parry CB, Brooks DM. Clinical trial of propranolol in post-traumatic neuralgia. *Pain*. 1982;14(3):283-292. doi:http://dx.doi.org/10.1016/0304-3959%2882%2990135-X

7. Bouhassira D, Danziger N, Attal N, Guirimand F. Comparison of the pain suppressive effects of clinical and experimental painful conditioning stimuli. *Brain*. 2003;126(5):1068-1078. doi:http://dx.doi.org/10.1093/brain/awg106

8. Colini Baldeschi G, Dario A, De Carolis G, et al. Peripheral Nerve Stimulation in the Treatment of Chronic Pain Syndromes From Nerve Injury: A Multicenter Observational Study. *Neuromodulation*. 2017;20(4):369-374. doi:http://dx.doi.org/10.1111/ner.12539

9. Ciaramitaro P, Mondelli M, Logullo F, et al. Traumatic peripheral nerve injuries: Epidemiological findings, neuropathic pain and quality of life in 158 patients. *J Peripher Nerv Syst*. 2010;15(2):120-127. doi:http://dx.doi.org/10.1111/j.1529-8027.2010.00260.x

10. Santana MVB, Bina MT, Paz MG, et al. High prevalence of neuropathic pain in the hand of patients with traumatic brachial plexus injury: A cross-sectional study. *Arq Neuropsiquiatr*. 2016;74(11):895-901. doi:http://dx.doi.org/10.1590/0004-282X20160149

11. Cheing GLY, Luk MLM. Transcutaneous electrical nerve stimulation for neuropathic pain. *J Hand Surg Am*. 2005;30(1):50-55. doi:10.1016/j.jhsb.2004.08.007

12. Sjolund KF, Belfrage M, Karlsten R, et al. Systemic adenosine infusion reduces the area of tactile allodynia in neuropathic pain following peripheral nerve injury: A multi-centre, placebo-controlled study. *Eur J Pain*. 2001;5(2):199-207. doi:http://dx.doi.org/10.1053/eujp.2001.0237

13. Nikolajsen L, Black JA, Kroner K, Jensen TS, Waxman SG. Neuroma removal for neuropathic pain: efficacy and predictive value of lidocaine infusion. *Clin J Pain*. 2010;26(9):788-793. doi:10.1097/AJP.0b013e3181ed0823

14. Taylor KS, Anastakis DJ, Davis KD, et al. Chronic pain and sensorimotor deficits following peripheral nerve injury. *Pain*. 2010;151(3):582-591. doi:http://dx.doi.org/10.1016/j.pain.2010.06.032

15. Kalliomäki J, Attal N, Jonzon B, et al. A randomized, double-blind, placebo-controlled trial of a chemokine receptor 2 (CCR2) antagonist in posttraumatic neuralgia. *Pain*. 2013;154(5):761-767. doi:10.1016/j.pain.2013.02.003

16. Novak CB, Anastakis DJ, Beaton DE, et al. Biomedical and psychosocial factors associated with disability after peripheral nerve injury. *J Bone Jt Surg - Ser A*. 2011;93(10):929-936. doi:http://dx.doi.org/10.2106/JBJS.J.00110

17. Goswami R, Anastakis DJ, Katz J, Davis KD. A longitudinal study of pain, personality, and brain plasticity following peripheral nerve injury. *Pain*. 2016;157(3):729-739. doi:http://dx.doi.org/10.1097/j.pain.0000000000000430

18. Witting N, Kupers RC, Svensson P, Jensen TS. A PET activation study of brush-evoked allodynia in patients with nerve injury pain. *Pain*. 2006;120(1-2):145-154. doi:http://dx.doi.org/10.1016/j.pain.2005.10.034

19. Lundborg G, Rosén B, Dahlin L, Danielsen N, Holmberg J. Tubular versus conventional repair of median and ulnar nerves in the human forearm: early results from a prospective, randomized, clinical study. *J Hand Surg Am*. 1997;22(1 CC-Child Health CC-Bone, Joint and Muscle Trauma CC-Neuromuscular):99‐106. doi:10.1016/S0363-5023(05)80188-1

20. Vollert J, Attal N, Baron R, et al. Quantitative sensory testing using DFNS protocol in Europe: an evaluation of heterogeneity across multiple centers in patients with peripheral neuropathic pain and healthy subjects. *Pain*. 2016;157(3):750-758. doi:https://dx.doi.org/10.1097/j.pain.0000000000000433

21. Novak CB, Mackinnon SE. Outcome following implantation of a peripheral nerve stimulator in patients with chronic nerve pain. *Plast Reconstr Surg*. 2000;105(6):1967-1972. http://ovidsp.ovid.com/ovidweb.cgi?T=JS&PAGE=reference&D=med4&NEWS=N&AN=10839393

22. Nashold BSJ, Goldner JL, Mullen JB, Bright DS. Long-term pain control by direct peripheral-nerve stimulation. *J Bone Joint Surg Am*. 1982;64(1):1-10. http://ovidsp.ovid.com/ovidweb.cgi?T=JS&PAGE=reference&D=med2&NEWS=N&AN=6976348

23. Gierthmuhlen J, Maier C, Baron R, et al. Sensory signs in complex regional pain syndrome and peripheral nerve injury. Baron R Binder A, Koroschetz J, Maier C, Richter H, Krumova EK, Westermann A, Tolle T, Berthele A, Sprenger T, Valet M, Munchen TU, Treede RD, Magerl W, Klein T, Birklein F, Geber C, Rolke R, Maihofner C, Azad SC, Beyer A, Huge V, Lauchart M, Birbaumer N GJ, ed. *Pain*. 2012;153(4):765-774. doi:https://dx.doi.org/10.1016/j.pain.2011.11.009

24. Chemnitz A, Dahlin LB. Consequences and adaptation in daily life - Patients’ experiences three decades after a nerve injury sustained in adolescence. *BMC Musculoskelet Disord*. 2013;14:252. doi:http://dx.doi.org/10.1186/1471-2474-14-252

25. Chen C, Tang P, Zhang X, et al. Treatment of soft-tissue loss with nerve defect in the finger using the boomerang nerve flap. *Plast Reconstr Surg*. 2013;131(1):44e-54e. doi:https://dx.doi.org/10.1097/PRS.0b013e3182729f5e

26. IRWIN MS, GILBERT SEA, TERENGHI G, SMITH RW, GREEN CJ. Cold Intolerance Following Peripheral Nerve Injury. *J Hand Surg Am*. 1997;22(3):308-316. doi:10.1016/s0266-7681(97)80392-0

27. Leung L. Pain catastrophizing: An updated review. *Indian J Psychol Med*. 2012;34(3):204-217. doi:10.4103/0253-7176.106012

28. Bouhassira D, Attal N, Alchaar H, et al. Comparison of pain syndromes associated with nervous or somatic lesions and development of a new neuropathic pain diagnostic questionnaire (DN4). *Pain*. 2005;114(1-2):29-36. doi:10.1016/j.pain.2004.12.010

29. Bouhassira D, Attal N, Fermanian J, et al. Development and validation of the Neuropathic Pain Symptom Inventory. *Pain*. 2004;108(3):248-257. doi:10.1016/j.pain.2003.12.024
