# Supplementary material for: A Quantitative Systematic Review of Clinical Outcome Measure Use in Peripheral Nerve Injury of the Upper Limb
Source: Neurosurgery. 2021 Mar 8;89(1):22–30. doi: 10.1093/neuros/nyab060 (PMC8203424; doi:10.1093/neuros/nyab060)
Supplement: nyab060_Supplemental_Files [file nyab060_supplemental_files.zip › SR Outcome Measures PNI.Supplementary Table 8.docx]

Supplementary Table 8: Neurotrophic measure outcome reporting

| Outcome Measures | No. of studies reporting outcome | Instrument | Metric | Time points |
| --- | --- | --- | --- | --- |
| Electrophysiology | 14 | 14 | 11 | 3 |
| Tinel’s Test | 2 | 2 | 2 | 1 |
| Central Nervous System (CNS) Imaging | 3 | 3 | 3 | 1 |
| Magnetic Resonance Neurography | 1 | 1 | 1 | 0 |
| End-organ imaging | 2 | 2 | 2 | 0 |
| Cognitive capacity testing | 1 | 1 | 0 | 0 |

Fourteen studies utilised electrophysiology as an outcome measure ^1–14^. In brachial plexus patients time points for assessment ranged from 4 weeks to 73 months after surgery, in mixed upper limb nerve injury patients time points for assessment ranged between 1 month to 8 years after injury. Only one study used electrophysiology in sensory nerve injuries and undertook assessments between 16-68 months after surgery.

Tinel’s test was used as an outcome measure in two studies: Lundborg et al. ^15^ conventionally assessed the distal most location of Tinel’s sign in the hand at 3,6 and 12-months after injury. Whereas Chen et al. graded Tinel’s test based on its degree of response to the percussive stimulus ranging from 1 (no tingling) – 4 (severe discomfort caused) ^16^.

Six studies used imaging modalities to assess regeneration. Three of these studies assessed central (cortical) changes during peripheral nerve regeneration. Taylor et al. ^8^ used functional magnetic resonance imaging (MRI) analysis; cortical thickness analysis and diffusion tensor imaging analysis after a sensory stimulus in brachial plexus injured patients at a mean of 4.8 years post-surgery; whereas Goswami et al. ^17^ used MRI to perform a cortical thickness analysis between 11-49 days after mixed upper limb nerve injury and Theuvenet et al. ^6^ used MRI to assess magnetoencephalography readings in patients with neuropathic pain at a mean of 5.4 years after mixed upper limb nerve injury. One study used MR neurography to assess regeneration of Oberlin (I) nerve transfers ^7^ with a mean follow-up of 4 years post-op and two studies used imaging to assess end-organ changes. Bosnjak et al. ^18^ assessed muscle cross-sectional area using MRI at a minimum of 9 years after surgery whilst Boonstra et al. ^3^ used computational tomography to assess muscle cross-sectional area (CSA) and muscle density, and also ultrasound scanning to assess muscle CSA.

Only one study used cognitive capacity testing to assess peripheral nerve regeneration. Mahmoud Aliloo et al. ^19^ utilised the Stroop Colour Test and the reaction time and ability to reproduce geometric drawings.

References

1. Krarup C, Rosen B, Boeckstyns M, Sorensen A, Lundborg G, Moldovan M. Sensation, mechanoreceptor, and nerve fiber function after nerve regeneration. *Ann Neurol*. 2017;82(6):940-950. doi:http://dx.doi.org/10.1002/ana.25102

2. Mackel R. Human cutaneous mechanoreceptors during regeneration: Physiology and interpretation. *Ann Neurol*. 1985;18(2):165-172. doi:http://dx.doi.org/10.1002/ana.410180202

3. Boonstra A, Van Weerden T, Eisma W, Pahlplatz V, Oosterhuis H. The effect of low-frequency electrical stimulation on denervation atrophy in man. *Scand J Rehabil Med*. 1987;19(3):127-134.

4. Zalis AW, Rodriquez AA, Oester YT, Mains DB. Evaluation of nerve regeneration by means of nerve evoked potentials. *J Bone Joint Surg Am*. 1972;54(6):1246-1250. http://ovidsp.ovid.com/ovidweb.cgi?T=JS&PAGE=reference&D=med1&NEWS=N&AN=4652055.

5. Daneyemez M, Solmaz I, Izci Y, et al. Prognostic factors for the surgical management of peripheral nerve lesions. *Tohoku J Exp Med*. 2005;205(3):269-275. doi:http://dx.doi.org/10.1620/tjem.205.269

6. Theuvenet PJ, de Munck JC, Peters MJ, van Ree JM, Lopes da Silva FL, Chen ACN. Anesthetic block of pain-related cortical activity in patients with peripheral nerve injury measured by magnetoencephalography. *Anesthesiology*. 2011;115(2):375-386. doi:http://dx.doi.org/10.1097/ALN.0b013e31821f6562

7. Frueh FS, Ho M, Schiller A, et al. Magnetic Resonance Neurographic and Clinical Long-Term Results After Oberlin’s Transfer for Adult Brachial Plexus Injuries. *Ann Plast Surg*. 2017;78(1):67-72. http://ovidsp.ovid.com/ovidweb.cgi?T=JS&PAGE=reference&D=emed18&NEWS=N&AN=618707587.

8. Taylor KS, Anastakis DJ, Davis KD. Cutting your nerve changes your brain. *Brain*. 2009;132(11):3122-3133. doi:https://dx.doi.org/10.1093/brain/awp231

9. Zhou J-M, Gu Y-D, Xu X-J, Zhang S-Y, Zhao X. Clinical research of comprehensive rehabilitation in treating brachial plexus injury patients. *Chin Med J (Engl)*. 2012;125(14):2516-2520. http://ovidsp.ovid.com/ovidweb.cgi?T=JS&PAGE=reference&D=med7&NEWS=N&AN=22882932.

10. Bilgin NG, Ozge A, Mert E, Yalcinkaya DE, Kar H. Importance of electromyography and the electrophysiological severity scale in forensic reports. *J Forensic Sci*. 2007;52(3):698-701. doi:http://dx.doi.org/10.1111/j.1556-4029.2007.00427.x

11. Sungpet A, Suphachatwong C, Kawinwonggowit V. One-fascicle median nerve transfer to biceps muscle in C5 and C6 root avulsions of brachial plexus injury. *Microsurgery*. 2003;23(1):10-13. http://ovidsp.ovid.com/ovidweb.cgi?T=JS&PAGE=reference&D=med4&NEWS=N&AN=12616512.

12. Becker M, Lassner F, Fansa H, Mawrin C, Pallua N. Refinements in nerve to muscle neurotization. *Muscle Nerve*. 2002;26(3):362-366. http://ovidsp.ovid.com/ovidweb.cgi?T=JS&PAGE=reference&D=med4&NEWS=N&AN=12210365.

13. Chu N-S, Chu EC. Conduction study of digital nerve function recovery following toe-to- digit transplantation and a comparison with digit-to-digit replantation. *Muscle and Nerve*. 1995;18(11):1257-1264. doi:http://dx.doi.org/10.1002/mus.880181107

14. Ko JH, Baltzer HL, Kircher MF, et al. Discussion: A Comparison of Outcomes of Triceps Motor Branch-to-Axillary Nerve Transfer or Sural Nerve Interpositional Grafting for Isolated Axillary Nerve Injury. *Plast Reconstr Surg*. 2016;138(2):265e-7e. doi:https://dx.doi.org/10.1097/PRS.0000000000002368

15. Lundborg G, Rosén B, Dahlin L, Danielsen N, Holmberg J. Tubular versus conventional repair of median and ulnar nerves in the human forearm: early results from a prospective, randomized, clinical study. *J Hand Surg Am*. 1997;22(1 CC-Child Health CC-Bone, Joint and Muscle Trauma CC-Neuromuscular):99‐106. doi:10.1016/S0363-5023(05)80188-1

16. Chen C, Tang P, Zhang X, et al. Treatment of soft-tissue loss with nerve defect in the finger using the boomerang nerve flap. *Plast Reconstr Surg*. 2013;131(1):44e-54e. doi:https://dx.doi.org/10.1097/PRS.0b013e3182729f5e

17. Goswami R, Anastakis DJ, Katz J, Davis KD. A longitudinal study of pain, personality, and brain plasticity following peripheral nerve injury. *Pain*. 2016;157(3):729-739. doi:http://dx.doi.org/10.1097/j.pain.0000000000000430

18. Bosnjak RF, Dolenc VV, Sepe A, Tindall SC, Demsar F. Force, fatigue, and the cross-sectional area of wrist extensor muscles after radial nerve grafting. *Neurosurgery*. 1992;31(6):1035-1042. http://ovidsp.ovid.com/ovidweb.cgi?T=JS&PAGE=reference&D=emed4&NEWS=N&AN=22370333.

19. MahmoudAliloo M, Bakhshipour A, Hashemi T, AR R, Hassan-Zadeh R. The correlation of cognitive capacity with recovery of hand sensibility after peripheral nerve injury of upper extremity. *NeuroRehabilitation*. 2011;29(4):373-379.
